# Stability of childhood maltreatment self-reports: a systematic review and meta-analysis

**DOI:** 10.1038/s44220-026-00677-7

**Published:** 2026-07-03

**Authors:** Oonagh Coleman, Maya Al-Jaber, Geethma Aponsu, Daniel Stahl, Andrea Danese

**Affiliations:** 1 Social, Genetic and Developmental Psychiatry Centre, Institute of Psychiatry, Psychology and Neuroscience, King’s College London, London, UK; 2 Department of Biostatistics and Health Informatics, King’s College London, London, UK; 3 Department of Child and Adolescent Psychiatry, Institute of Psychiatry, Psychology and Neuroscience, King’s College London, London, UK; 4 National and Specialist CAMHS Clinic for Trauma, Anxiety, and Depression, South London and Maudsley NHS Foundation Trust, London, UK

**Keywords:** Psychology, Risk factors

## Abstract

Research and clinical practice on childhood maltreatment largely rely on retrospective self-reports. However, self-reports are often considered unreliable due to concerns about memory biases and shifting subjective appraisals. Here, in a meta-analysis of 49 studies including *n* = 38,332 individuals followed for an average of 2.4 years (range = 2 months to 12 years), we found that retrospective self-reports of maltreatment are overall highly stable (*r* = 0.79). However, stability was lower in population-representative samples than in clinical or convenience samples, for neglect compared with abuse, and in children compared with adults. In children, but not adults, stability declined with longer follow-up. These findings challenge the view that retrospective self-reports are inherently unstable, although further research is needed to investigate long-term stability. Reshaping trauma-related appraisals may require deliberate intervention and might be most effective during childhood when memories appear more malleable.

## Abstract (web summary)

In this meta-analysis of 49 studies, the authors find that self-reports of childhood maltreatment are generally stable, challenging assumptions of inherent unreliability and suggesting that traumatic memories may be more easily targeted by intervention during childhood.

## Main

Childhood maltreatment is a key modifiable risk factor for poor mental health^1^. Childhood maltreatment can be measured through either prospective measures, typically derived from informant reports and observations, or retrospective self-reported measures, which identify largely non-overlapping groups^2^. Nevertheless, much of our understanding of its long-term effects and underlying mechanisms comes from studies comparing groups of individuals with versus without retrospective self-reports of maltreatment, which are quicker, easier and cheaper to collect than prospective measures^3^. Retrospective self-reports also show stronger associations with psychopathology than prospective measures, regardless of whether they are corroborated by prospective evidence^4,5^. But how stable is this method of classification over time?

Instability in childhood maltreatment reports could pose serious threats to both research and practice. Concerns about the low temporal stability of retrospective self-reports stem from their reliance on memory and subjective interpretation, which can be influenced by cognitive biases, emotional states and reappraisals over time^3,6–9^. Furthermore, the instability of childhood maltreatment reports may be compounded by normative developmental changes in autobiographical memory^10–12^. From a neurobiological perspective^10,13^, early life memories depend heavily on an immature hippocampal system, resulting in weaker encoding and inefficient consolidation—a combination that contributes to rapid forgetting and childhood amnesia. In contrast, with age, the hippocampus matures, supporting stronger episodic encoding, and hippocampal–cortical connectivity increases substantially. These expanding connections with distributed cortical networks (supported by schema-based organization and repeated retrieval or narrative rehearsal) enhance systems consolidation and may lead to greater long-term retention and reduced forgetting in later life. Low stability of retrospective maltreatment measures contributes to measurement error and misclassification^14^. In turn, misclassification could lead researchers to underestimate associations between maltreatment and outcomes, observe inconsistent findings across studies and struggle to identify underlying mechanisms by weakening mediation and moderation tests. Similarly, misclassification could lead legal, social care and clinical practitioners to provide inadequate or inappropriate interventions to mitigate the impact of childhood maltreatment.

Several studies^15,16^ have empirically tested the stability of retrospective self-reports with inconsistent results, highlighting the need for a quantitative summary and a better understanding of the heterogeneity in the literature. Several factors may explain the inconsistencies. First, the maltreatment type (for example, abuse versus neglect) may be relevant, as experiences can differ in their emotional salience and thus be differentially appraised and recalled over time^17^. Second, the sample characteristics: convenience samples may show higher stability than population-representative samples because of the effects of self-selection^18,19^ or differential attrition over time^20^; clinical samples are a pertinent example, in which participants with psychiatric diagnoses may show higher stability of recall than healthy controls due to cognitive mechanisms linked to psychopathology (for example, mood congruency effects, rumination, causal search biases or increased reflection on the past in therapy)^21–23^. Third, measurement type (for example, interviews versus questionnaires), as interviews may reduce measurement error by allowing clarifications but are also more prone to social desirability bias^7,24^, whereas questionnaires may be more prone to measurement error but offer privacy that may enhance consistency^25^. Fourth, demographic factors: some evidence suggests that female individuals may show higher report stability than male individuals^16^; younger age may also be associated with lower stability, as children and adolescents may initially struggle to identify maltreatment, have less developed memory processes and continue developing their identity and life narratives^12,26–28^. Fifth, the follow-up interval may be a factor, as longer intervals could be associated with lower stability, consistent with the forgetting curve^29,30^.

## Results

### Search results

The systematic search identified 49 studies examining the stability of self-reports of child maltreatment^15,16,31–77^ (Table 1 and Supplementary Fig. 2). The studies were based on 49 cohorts (although 1 study used 2 different cohorts, and 1 cohort was used by 2 studies). There were 92,807 individuals assessed at baseline and 38,332 of these individuals were retained at follow-up, with an average attrition rate of 59%. Participants were 60.4% female, the average age at baseline assessment was 32.2 years (range = 13.2–68.5, median = 33.0), the average age at follow-up assessment was 34.4 years and the average follow-up interval was 2.4 years (123.6 weeks; range = 2 months to 12 years). The study quality assessment is described in Supplementary Table 1.

**Table 1:** Study characteristics

Source	Cohort	Country	Sample size at baseline	Sample size at follow-up	Loss to attrition (%)	Sex (% female)	Sample type	Measure type	Maltreatment type	Age at baseline	Age at follow-up	Time interval (weeks)
Aalsma et al.^31^	Orr STD	USA	444	217	51	83	Non-representative	Questionnaire	Sexual abuse	17	17	20.42
Bernstein et al.^32^	Ruggiero Addiction	USA	286	40	86	14.7	Clinical	Questionnaire	Maltreatment, emotional abuse, physical abuse, emotional neglect, physical neglect and sexual abuse	40.2	40.2	15.6
Bifulco et al.^33^	Jacobs Islington	UK	303	111	63	100	Clinical	Questionnaire	Sexual abuse, physical abuse, emotional neglect and physical neglect	34.5	36.5	104.29
Boisselier and Soubelet^34^	Soubelet MACE	France	402	50	87.6	72	Non-representative	Questionnaire	Physical abuse, emotional abuse, emotional neglect and maltreatment	34.28	25.28	55
Breton et al.^35^	MLSFH ACE	Malawi	2,061	1,878	8.89	49	Population representative	Questionnaire	Physical neglect, sexual abuse, physical abuse, emotional abuse and emotional neglect	13.21	16.51	172.2
Cammack et al.^36^	Newport WMHP	USA	1,975	244	87.6	100	Clinical	Questionnaire	Sexual abuse, physical abuse, emotional abuse, emotional neglect and physical neglect	32.7	35.2	140.7
Cay et al.^37^	Shinn Schizophrenia	USA	128	128	0	54.69	Clinical	Questionnaire	Sexual abuse, physical abuse, emotional abuse, emotional neglect, physical neglect and maltreatment	25	26.6	85.17
Chatziioannidis et al.^38^	Bozikas SSP	Greece	63	46	27	30.16	Clinical	Questionnaire	Sexual abuse, physical abuse, physical neglect and emotional abuse	40.44	40.75	13
Colman et al.^15^	NPHS	Canada	14,442	7,466	48	54.12	Population representative	Questionnaire	ACEs	NA	NA	624
Dube et al.^39^	Anda HAC	USA	9,508	658	93	51	Non-representative	Questionnaire	Physical abuse, sexual abuse and emotional abuse	64	65.7	86.6
Durrett et al.^40^	Silk_PBD	USA	421	170	59.6	53.39	Clinical	Questionnaire	Sexual abuse and physical abuse	18	20	104
Fergusson et al.^41^	CHDS	New Zealand	1,265	983	22.5	49.8	Population representative	Interview	Sexual abuse and physical abuse	18	21	156.4
Fisher et al.^42^	ÆSOP	UK	84	30	64.3	43.3	Clinical	Questionnaire	Sexual abuse, physical abuse, emotional neglect and physical neglect	29	36	365
Frampton et al.^43^	EmbrACE	Canada	284	284	0	74.6	Non-representative	Questionnaire	ACEs	40.96	40.96	13
Friedrich et al.^44^	Zinmeister Olmsted	USA	920	610	34	53	Population representative	Questionnaire	Sexual abuse, physical abuse and emotional abuse	40.3	42	86.6
Goldberg and Freyd^45^	ECSC	USA	736	689	6	57.6	Non-representative	Questionnaire	Sexual abuse, physical abuse, emotional abuse and ACEs	58.5	61.5	156.43
Goldsmith et al.^46^	DePrince Psychology Course	USA	185	96	48.1	68.1	Non-representative	Questionnaire	Sexual abuse, physical abuse, emotional abuse and maltreatment	19.21	20.71	78.2
Goltermann et al.^47^	MACS	Germany	768	598	22.1	61.7	Clinical	Questionnaire	Maltreatment, emotional abuse, physical abuse, sexual abuse, emotional neglect and physical neglect	35.41	37.41	110.93
Goltermann et al.^47^	MNC	Germany	261	168	35.6	61.7	Clinical	Questionnaire	Maltreatment, emotional abuse, physical abuse, sexual abuse, emotional neglect and physical neglect	40.98	42.98	116.63
Hagborg et al.^48^	LoRDIA	Sweden	1174	603	48.6	52	Population representative	Questionnaire	Sexual abuse, physical abuse, physical neglect, emotional abuse and emotional neglect	14.5	15.5	52.14
Hardt et al.^49^	Egle MSBI	Germany	100	100	0	76	Non-representative	Interview	Physical abuse and sexual abuse	40.2	42.4	114.7
Hoeboer et al.^50^	IMPACT	The Netherlands	149	100	32.9	76.5	Clinical	Questionnaire	Sexual abuse, physical abuse, physical neglect, emotional abuse and emotional neglect	36.9	37.4	26
Hörberg et al.^51^	Ramklint Uppsala	Sweden	143	42	70.6	75.5	Clinical	Questionnaire	Sexual abuse, physical abuse and emotional abuse	23.4	23.6	11.2
Hosang et al.^52^	BADGE	UK	85	53	37.6	72.9	Clinical	Questionnaire	Sexual abuse, physical abuse, physical neglect, emotional abuse, emotional neglect and maltreatment	51.7	61.7	521.43
Kerbage et al.^53^	Olié suicide	France	533	164	69	64.6	Clinical	Questionnaire	Sexual abuse, physical abuse, physical neglect, emotional abuse, emotional neglect and maltreatment	43.4	43.9	26
Kremers et al.^54^	Spinhoven BPD	The Netherlands	84	50	40	90	Clinical	Interview	Sexual abuse, physical abuse, emotional abuse and maltreatment	31.1	33.5	117.32
McKinney et al.^55^	Caetano couples	USA	2,542	2,256	11.2	50.7	Population representative	Interview	Physical abuse	41.4	46.4	260.7
Mersky et al.^56^	Topitzes FFHV	USA	1,241	83	93	100	Non-representative	Interview	Sexual abuse, physical abuse, emotional abuse, physical neglect, emotional neglect and ACEs	24.2	24.95	39.43
Mesquita and Maia^57^	Maia DSM	Portugal	120	34	71.7	64.7	Clinical	Interview	Sexual abuse, physical abuse, emotional abuse and physical neglect	47.18	48.18	52.14
Naicker et al.^58^	Bt20+	South Africa	3,273	1,595	51	0	Population representative	Questionnaire	Sexual abuse, physical abuse and emotional abuse	15	22	365
Nelson et al.^59^	Childhood trauma study	Australia	6,265	1,532	75.5	0	Non-representative	Interview	Sexual abuse and physical abuse	37	44.16	373.34
Okeke et al.^60^	Add_Health	USA	151,197	12,438	18.2	54.82	Population representative	Questionnaire	Sexual abuse	21.9	29.9	312.86
Paivio^61^	Paivio EFT-AS	Canada	44	33	25	81.8	Clinical	Questionnaire	Sexual abuse, emotional neglect, physical neglect and maltreatment	37	37	26
Paivio and Cramer^62^	Cramer Saskatchewan	Canada	429	87	79.7	65	Non-representative	Questionnaire	Sexual abuse, physical abuse, emotional abuse, emotional neglect, physical neglect and maltreatment	19	19	9
Park^63^	Park Seoul	Korea	86	51	40.6	NA	Clinical	Questionnaire	Physical abuse and emotional abuse	14.5	14.66	8
Peng et al.^64^	Yu China	China	20,951	1,389	93.4	50.4	Population representative	Questionnaire	Sexual abuse, physical abuse, emotional neglect, physical neglect and maltreatment	15.27	15.77	26
Pereira Da Silva and Da Costa Maia^65^	Maia obese	Portugal	30	30	0	66.67	Non-representative	Questionnaire	Sexual abuse, physical abuse, emotional abuse, physical neglect and emotional neglect	39.17	40.17	52
Pinto et al.^66^	Maia CPS	Portugal	136	79	42	48.1	Non-representative	Questionnaire	Sexual abuse, physical abuse, emotional abuse, physical neglect and emotional neglect	17	17	26
Plaza et al.^67^	Garcia-Esteve postpartum depression	Spain	329	20	94	100	Non-representative	Questionnaire	Sexual abuse, physical abuse, emotional abuse and maltreatment	31.9	31.9	13
Racine et al.^68^	AOB/AOF	Canada	3,388	19,112	50.8	100	Non-representative	Questionnaire	Sexual abuse, physical abuse, emotional abuse and maltreatment	30.89	33.89	161.43
Shannon et al.^69^	Mulholland BD	Ireland	60	39	35	51.2	Clinical	Questionnaire	Sexual abuse, physical abuse, emotional abuse, emotional neglect and physical neglect	49.1	51.2	78.2
Simpson et al.^70^	Bendall EPPIC	Australia	94	54	38.3	40.7	Clinical	Questionnaire	Maltreatment	20.8	20.8	14
Smith et al.^71^	Checkley London	UK	108	30	72	52.8	Clinical	Questionnaire	Sexual abuse and physical abuse	44	47	156.43
Spinhoven et al.^72^	Spinhoven BPD	The Netherlands	339	229	32	52.8	Clinical	Questionnaire	Sexual abuse and physical abuse	37.89	38.89	52.14
Teicher and Parigger^73^	Parigger McLean	USA	407	75	81.6	61.4	Non-representative	Questionnaire	Sexual abuse, physical abuse, emotional abuse, emotional neglect and physical neglect	23.3	23.3	9.5
Wielaard et al.^74^	NESDO	The Netherlands	510	277	45.6	65.3	Clinical	Interview	Sexual abuse, physical abuse, emotional abuse and emotional neglect	68.5	74.5	312.86
Xiang et al.^75^	Long Changsha	China	50	35	30	57.14	Clinical	Questionnaire	Sexual abuse, physical abuse, emotional abuse, emotional neglect and physical neglect	17.91	18.76	48.92
Yancura and Aldwin^16^	DLS	USA	577	577	0	53.8	Non-representative	Questionnaire	Physical abuse	42.24	48.24	260
Zanotti et al.^76^	Cromer NCAA	USA	141	141	0	52.3	Non-representative	Questionnaire	ACEs	19.55	20.55	49.57
Zhang et al.^77^	Chen Chinese CTQ	China	188	188	0	66	Non-representative	Questionnaire	Sexual abuse, physical abuse, emotional abuse, emotional neglect, physical neglect and maltreatment	18.4	19.4	52

NA, not available.

### Stability of self-reports of maltreatment

**Fig. 1:**
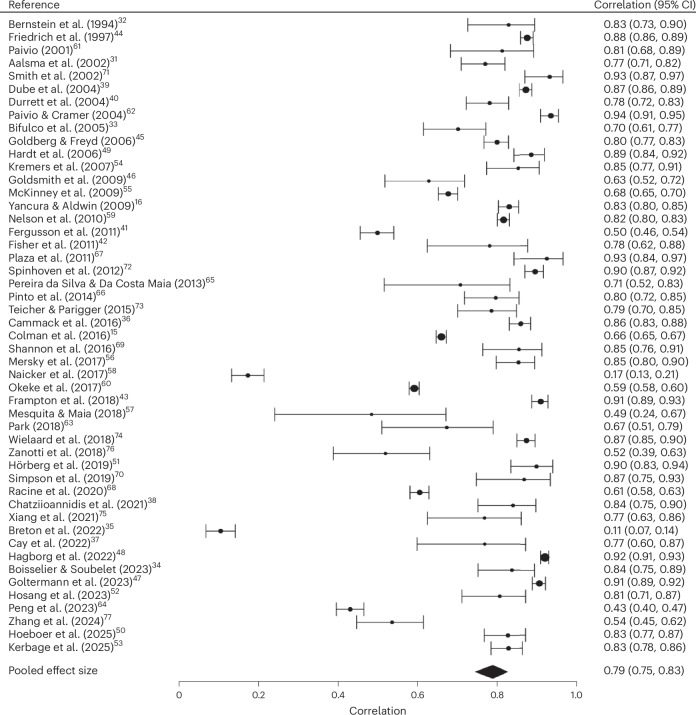
Forest plot depicting stability estimates for each study. The overall pooled effect size was 0.79. Points represent the correlation estimate for each study and horizontal error bars represent the corresponding 95% confidence intervals. For clarity of presentation, the forest plot shows a single effect size per study (reflecting the mean of all individual effect sizes obtained from each study). The mean effect size per study and its variance were calculated using the MAd package in R (R Foundation). Data from refs. ^15,16,31–77^.

The overall meta-analytic estimate for the stability of self-reports of childhood maltreatment based on 235 effect sizes was *r* = 0.79 (95% confidence interval (CI), 0.75, 0.83), with heterogeneity *I*^2^ = 98.69 (Fig. 1). When excluding the 34 effect sizes from 11 studies estimated from the calibration model, the meta-analytic findings were very similar (*r* = 0.79; 95% CI, 0.74, 0.83).

The results were not biased by small study effects (that is, publication bias) (*P* = 0.561) (Supplementary Fig. 3). The results were also not biased by undue effects of individual effect sizes (estimates from leave-one-out analyses ranged between 0.788 and 0.793), individual studies (range = 0.785–0.800; Supplementary Fig. 4) or individual cohorts (range = 0.785–0.800; Supplementary Fig. 5).

**Fig. 2:**
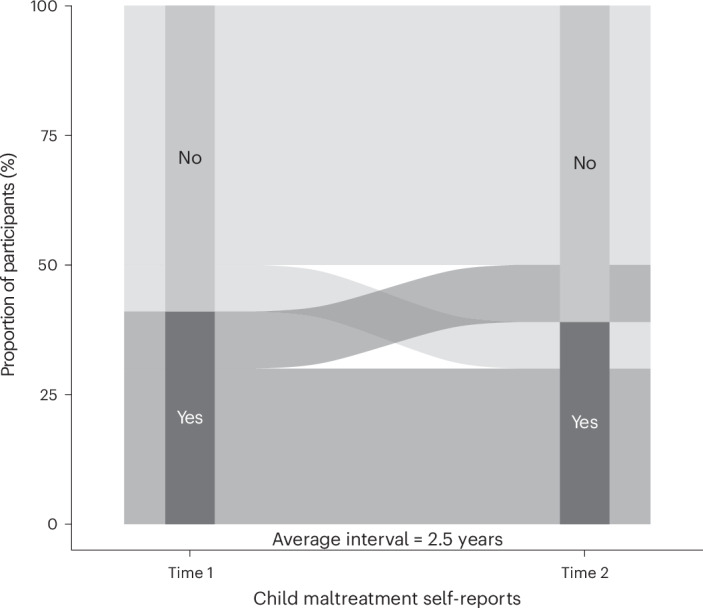
Sankey plot showing the estimated proportion of participants changing their response from time 1 to time 2. Prevalence rates at baseline and follow-up were derived from *k* = 5 population-representative studies^15,35,44,58,60^ reporting 2 × 2 contingency tables. We extracted the proportion of participants reporting maltreatment at each time point, averaging 41% at time 1 and 39% at time 2. Using these prevalence estimates and a correlation of *r* = 0.79, we iteratively searched for the combination of response shifts that produced the observed change in prevalence and best approximated the target correlation. This yielded an estimate of approximately 20% of participants changing their response: 11% shifting from reporting maltreatment at baseline to not reporting it at follow-up, and 9% newly reporting maltreatment at follow-up despite not endorsing it at baseline.

To illustrate the overall stability estimate, we also computed the approximate proportion of participants changing their responses between time points, based on the observed prevalence rates and correlation from population-representative studies. This indicated that approximately 20% of participants changed their responses, with 11% shifting from reporting maltreatment at baseline to not reporting it at follow-up and 9% shifting from not reporting maltreatment at baseline to reporting it at follow-up (Fig. 2).

### Predictors of heterogeneity in the stability of self-reports of maltreatment

We next examined various possible sources of the high heterogeneity in the stability estimate (Table 2).

**Table 2:** Moderation analyses

Moderator	Number of studies	Participants (*N*)	Number of effect sizes	*r* (95% CI)	*Q* for moderation	*P* value	Variance explained by moderator (%)
Type of maltreatment					25.83	**<0.0001**	1.61
ACEs	5	8,371	7	0.81 (0.70, 0.88)			
Emotional abuse	32	11,755	40	0.79 (0.74, 0.84)			
Emotional neglect	22	5,029	27	0.75 (0.67, 0.81)			
Maltreatment	16	4,977	23	0.81 (0.75, 0.86)			
Physical abuse	42	17,723	54	0.81 (0.77, 0.85)			
Physical neglect	24	6,221	31	0.69 (0.60, 0.76)			
Sexual abuse	42	27,523	53	0.81 (0.76, 0.85)			
Measure type					0.07	0.793	−1.48
Interview	8	5,315	32	0.78 (0.65, 0.86)			
Questionnaire	41	33,067	203	0.79 (0.75, 0.83)			
Sampling					11.54	**0.003**	12.11
Population representative	9	29,218	30	0.62 (0.46, 0.74)			
Convenience or non-representative	18	6,526	78	0.81 (0.74, 0.86)			
Clinical	22	2,588	127	0.83 (0.78, 0.87)			
Sex distribution	48	38,281	233	0.00 (0.00, 0.00)	3.2	0.074	1.66
Baseline age	48	30,866	234	0.01 (0.00, 0.02)	7.02	**0.008**	7.70
Follow-up age	48	30,866	234	0.01 (0.00, 0.01)	4.59	**0.032**	4.61
Time interval	49	38,332	235	0.00 (0.00, 0.00)	2.96	0.085	2.81
Attrition rate	49	38,332	235	0.00 (0.00, 0.01)	2.05	0.152	2.29
Quality	49	38,332	235	−0.08 (−0.19, 0.03)	1.97	0.161	1.15

For continuous moderators (baseline age, follow-up age, time interval, attrition rate and study quality), the coefficient (*r*) represents the estimated change in the test–retest correlation for each 1-unit increase in the moderator. Bold font indicates statistical significance.

Stability was moderated by the maltreatment type (*Q* for moderation = 25.83; *P* < 0.0001), with lower stability estimates for physical neglect relative to physical abuse, sexual abuse, emotional abuse, maltreatment and adverse childhood experiences (ACEs), and lower stability estimates for emotional neglect relative to physical abuse and sexual abuse. In a post hoc analysis, we also found that stability estimates were significantly lower for overall measures of neglect (number of studies (*k*) = 25; *r* = 0.72; 95%CI, 0.65, 0.78) than abuse (*k* = 45; *r* = 0.81; 95% CI, 0.76, 0.84; *Q* for moderation = 19.94; *P* < 0.0001).

Stability was also moderated by the type of sample used (*Q* for moderation = 11.54; *P* = 0.003), with population-representative samples showing significantly lower stability estimates compared with non-representative convenience samples or clinical samples. The overall meta-analytic estimate was not moderated by the assessment method (interview versus questionnaire). Furthermore, a post hoc analysis of studies using questionnaires showed a non-significant difference in stability estimates between those with single-item measures (*k* = 6; *r* = 0.73; 95% CI, 0.61, 0.82) and those with multi-item measures (*k* = 37; *r* = 0.81; 95% CI, 0.60, 0.92; *Q* for moderation = 3.03; *P* = 0.08).

Baseline and follow-up age moderated the effect size estimate, with stability increasing with age (baseline age, *Q* for moderation = 7.02, *P* = 0.008; follow-up age, *Q* for moderation = 4.59, *P* = 0.032). Visual inspection (Supplementary Fig. 6) indicated that the stability did not vary linearly with baseline age, with notably lower stability estimates among younger participants. The addition of a logarithmic term improved model fit compared with a linear model in the meta-regression (likelihood ratio test *χ*^2^ = 5.501, *P* = 0.019) (Supplementary Fig. 7). Furthermore, we found that studies with baseline ages between 0 and 18 years (*k* = 8; *r* = 0.65; 95% CI, 0.49, 0.77) had lower stability compared with studies with baseline ages greater than 18 years (*k* = 40; *r* = 0.81; 95% CI, 0.77, 0.85; *Q* for moderation = 6.88; *P* = 0.009). We also repeated this moderation analysis using alternative cut-off points to define childhood and adolescence, and observed a similar pattern of results when applying cut-offs at ages 21 and 24 (Supplementary Table 2).

**Fig. 3:**
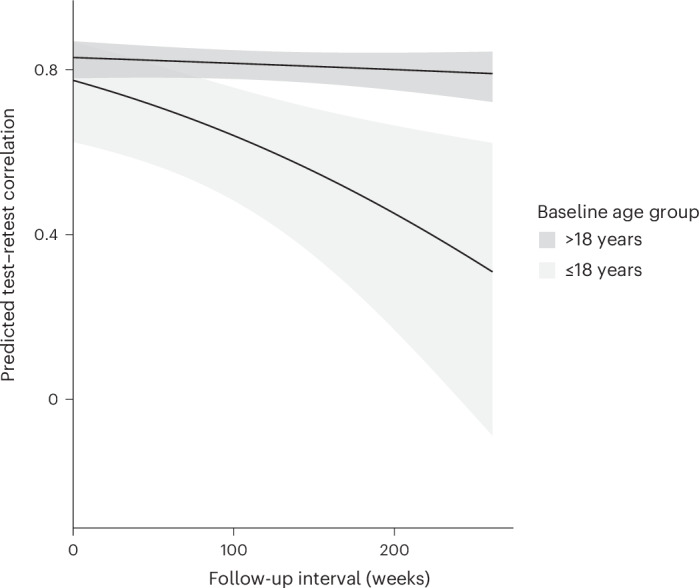
Interaction between baseline age and follow-up interval predicting stability. Predictions from an interaction model between baseline age (age 18 cut-off) and follow-up interval, inverse-transformed to the correlation scale for 0–261 weeks (0–5 years), showing that stability decreased over time for participants under 18 but remained stable over time for adults. The lines represent the predicted correlation estimates from the interaction model for adults and participants under 18 across follow-up intervals, and the gray shaded areas represent the corresponding 95% confidence intervals around these predictions.

Stability was not significantly moderated by time interval (*Q* for moderation = 2.96, *P* = 0.085) (Table 2). We further explored whether the associations between time interval and stability differed depending on baseline age. We found a significant interaction effect (unstandardized meta-regression coefficient (*b*) = 0.002; 95% CI, 0.0001, 0.005; *P* = 0.04), indicating that stability decreased over time for participants under 18, while it remained stable over time for adults (Supplementary Table 3). On the basis of predictions from this model, stability was *r* = 0.59 for those under 18 and *r* = 0.81 for adults at 2.5 years, and *r* = 0.31 and *r* = 0.79, respectively, at 5 years (Fig. 3). We repeated these analyses for the alternative age cut-offs for childhood and adolescence, and found a comparable pattern of results (Supplementary Figs. 8 and 9 and Supplementary Table 3).

We considered that lower stability for participants under 18 might reflect new exposures rather than changes in self-reports over time. In a sensitivity analysis excluding 2 studies that included participants under 18 but did not explicitly specify the same reporting period across time points^35,63^, we found a similar meta-analytic estimate (*r* = 0.80; 95% CI, 0.76, 0.83). The moderation analyses focusing on baseline age and the interaction between baseline age and follow-up interval yielded a similar patterns of results, although the results did not reach statistical significance (*P* = 0.10 for the moderation model, *P* = 0.09 for the interaction model; Supplementary Fig. 10 and Supplementary Tables 4 and 5). However, when we repeated these analyses using the broader age cut-offs for childhood and adolescence, we observed significant moderation and interaction effects (Supplementary Figs. 11 and 12 and Supplementary Tables 4 and 5).

We also conducted a post hoc moderation analysis to examine whether stability estimates differed by effect size type. We found significant differences (*Q* for moderation = 14.1, *P* = 0.001), with greater stability in studies using intraclass correlation coefficients (ICCs) (*k* = 17; *r* = 0.86; 95% CI, 0.82, 0.89) than in those using tetrachoric (*k* = 24; *r* = 0.75; 95% CI, 0.69, 0.81) and Pearson’s or Spearman’s correlations (*k* = 10; *r* = 0.72; 95% CI, 0.59, 0.81).

The meta-analytic estimate was not significantly moderated by sex distribution, attrition rate or the study quality.

Within the subset of studies with clinical samples, stability was not moderated by the proportion of individuals with clinical diagnoses, the type of psychopathology or the presence of an intervention between baseline and follow-up (Table 3; see Supplementary Table 6 for a description of studies that included an intervention). Similar results were observed in sensitivity analyses restricted to studies that did not include healthy volunteers (Supplementary Table 7).

**Table 3:** Moderation analyses for the clinical subgroup

Moderator	Number of studies	Participants (*N*)	Number of effect sizes	*r* (95% CI)	*Q* for moderation	*P* value	Variance explained (%)
Proportion diagnosis	22	2,588	127	0.00 (0.00, 0.00)	0.1	0.754	−1.31
Diagnosis					0.42	0.999	−17.38
Addiction	1	100	6	0.83 (0.56, 0.94)			
Bipolar	1	40	17	0.80 (0.68, 0.88)			
Depression	3	109	32	0.83 (0.73, 0.89)			
General psychopathology	5	1,369	22	0.83 (0.72, 0.89)			
Personality disorder	5	383	8	0.85 (0.72, 0.92)			
Psychosis	3	399	37	0.82 (0.73, 0.88)			
PTSD	5	205	5	0.83 (0.56, 0.94)			
Intervention					0.3	0.584	0
Intervention	6	570	28	0.84 (0.78, 0.88)			
No intervention	17	2,018	99	0.82 (0.78, 0.85)			

## Discussion

This systematic review and meta-analysis of 49 studies including 38,332 individuals found that retrospective self-reports of childhood maltreatment were stable over an average follow-up period of 2.4 years. Despite the high stability of the reports, 1 in 5 (20%) participants changed their responses over the observational period (at follow-up, 11% omitted reports made at baseline and 9% provided new reports that were not made at baseline; Fig. 2). The overall meta-analytic effect size of *r* = 0.79 is consistent with the high long-term stability observed for the self-reports of broader measures of childhood experiences^78^ and the stability of key psychological constructs, such as cognition^79^. The overall meta-analytic estimate showed high heterogeneity and should therefore be interpreted with caution. To better understand this heterogeneity, we undertook moderation analyses, which identified factors that influence stability and important gaps in our current knowledge.

First, sample type moderated stability. Population-representative samples showed significantly lower stability than both non-representative convenience samples and clinical samples—differences that might emerge for several reasons. For example, measure characteristics might have differed across sample types: larger, population-representative samples may use briefer, less detailed assessments, increasing the likelihood of reporting inconsistency. However, we found limited support for this hypothesis. We did not find that the use of interviews versus questionnaires moderated stability estimates, and a post hoc analysis indicated that the difference in stability between studies using multi-item versus single-item questionnaires was not statistically significant (trend).

Participant characteristics may explain stability differences between sample types. Participants in population-representative samples, who do not self-select into research, may have less survey experience, weaker topic knowledge, lower certainty or lower introspection about their experiences, reducing report stability^18^. For example, in the UK Biobank study^19^, participants with lower participation probability made more self-report errors in supposedly time-invariant variables and errors increased after adjusting for participation bias, suggesting greater stability in non-representative samples.

Clinical samples also showed higher stability than population-representative samples, likely due to cognitive mechanisms linked to psychopathology. For example, the mood congruency model suggests that negative memories are more easily recalled in low mood states^21,80^, while rumination—common in depression, anxiety and post-traumatic stress disorder (PTSD)—may enhance recall over time through repeated rehearsal^22^. Greater reflection during treatment may further enhance memory accessibility. Causal search bias, where individuals reinterpret past experiences to explain their distress or difficulties, could also increase consistency by reinforcing negative interpretations of memories over time^23^. Finally, more severe or chronic maltreatment experiences may heighten both psychopathology risk and recall consistency over time^17^. Of note, stability did not vary by psychopathology type, suggesting that transdiagnostic rather than disorder-specific mechanisms may be driving higher stability in clinical populations.

Second, maltreatment type moderated stability. Reports of neglect showed lower stability than reports of abuse or broader measures of maltreatment. This aligns with previous evidence that retrospective reports of neglect also show lower agreement with prospective measures of neglect compared with other maltreatment types^2^, suggesting greater variability in retrospective recall may partly explain this pattern. On the one hand, threatening experiences, such as abuse, may be more strongly encoded in memory and persist unscathed^17,81^. On the other hand, neglect involves the absence of actions or resources, rather than the occurrence of more tangible events that typically serve to anchor memories^82^, and its definition might vary over time as individuals and societies change their interpretations of what constitutes a child’s ‘basic needs’ and adequate caregiving practices^83^.

Third, age moderated stability. Report stability was significantly lower among participants who were first asked to recall maltreatment in childhood than those who were first asked to recall it in adulthood. Furthermore, stability significantly decreased as a function of follow-up duration in participants who first recalled maltreatment in childhood but not in those who first recalled it in adulthood. These findings should be interpreted with caution, as a sensitivity analysis excluding 2 studies involving under-18 participants, where new experiences might have affected reporting, showed similar patterns but weaker evidence. However, significant moderation and interaction effects were observed when using broader age cut-offs to define childhood and adolescence versus adulthood, even after excluding these 2 studies, suggesting that the non-significant finding at age 18 may reflect limited statistical power due to the smaller number of studies in this subgroup. The consistent effects across broader age definitions support the interpretation that younger participants show less stable reporting, particularly over longer periods.

These findings are consistent with a large body of literature^11,12,26,27,84,85^ on the developmental progression of autobiographical memory and life narratives, which become increasingly stable and coherent from childhood into adulthood thanks to social–cognitive development involving temporal sequencing, causal and thematic coherence, and the cultural concept of biography. Children’s and adolescents’ memories may be more malleable and susceptible to reinterpretation because of new knowledge, evolving life narratives and sense of self, and shifting social contexts^26,28^. In contrast, adults’ memories may be more stable over time because of repeated rehearsal and integration into a coherent life narrative^27,85^. Of note, similar patterns of moderation by age are also observed for highly stable traits, such as cognitive abilities and personality^79,86^, reflecting greater overall malleability of psychological processes in childhood and adolescence.

The findings should be interpreted in the context of some limitations. First, the number of population-representative samples was small, limiting the generalizability of the findings. Nevertheless, stability was relatively high (*r* = 0.62) even among population-representative samples. The findings should also be interpreted within the context of research studies similar to those included in the meta-analyses and may not generalize to other contexts (for example, to disclosures that reach the attention of authorities). Specifically, the included studies relied on research interviews or questionnaires completed in confidential research settings, which may elicit different reporting patterns than official or forensic disclosures made in the context of police interviews, investigations by child protection services or legal proceedings, where additional motivational factors may influence reporting.

Second, attrition rates were high, with an average loss of more than half the participants between baseline and follow-up. While no significant effect was detected in our moderation analysis, attrition could still introduce bias^20^, particularly because studies did not consistently report whether participants lost at follow-up systematically differed from those retained. For example, we found that stability is greatest in individuals with mental health problems (clinical samples), and greater attrition among individuals with mental health problems might reduce stability estimates in population samples.

Third, the average follow-up interval was 2.4 years—a relatively short time frame in which memories for major childhood events may be less likely to change. It will be important to test how stability varies over longer intervals, particularly in light of findings that even highly stable traits, such as personality, show greater decline in stability over longer intervals^86^.

Fourth, the number of studies with participants under 18 was limited, and in 2 studies^35,63^ it was unclear whether changes reflected shifts in self-reports over time or the occurrence of new experiences between assessments. Further research is needed to examine the stability of self-reports in children and adolescents, with a focus on ensuring consistency in the reporting period across assessments.

Fifth, the preregistered search strategy did not include databases from adjacent fields such as nursing, social work, education or criminology, where other relevant studies on maltreatment and trauma assessment might also be found. Moreover, it focused on core maltreatment terms and may have missed studies using broader descriptors such as violence or victimization. Future reviews could expand searches to include these terms and interdisciplinary databases to ensure more comprehensive coverage.

Sixth, we found differences in estimates by effect size type, ICC estimates showing significantly higher stability compared with tetrachoric or Pearson and Spearman correlations. However, although the differences are statistically significant, the effect sizes are qualitatively similar (all large) and support the same conclusion of high stability.

Finally, due to a lack of available data, we were unable to examine in detail the effects of several other factors on self-report stability, including event characteristics (for example, age at maltreatment occurrence, severity and duration), ethnicity, socioeconomic status, personality, cognitive abilities, changes in psychopathology^15^ or therapeutic intervention (only six studies included an intervention, only one of which was trauma-focused, that is, specifically targeting maladaptive cognitive appraisals or memory processes^50^) (Supplementary Table 6).

Our findings have important implications for research and practice. The high stability of retrospective maltreatment reports over an average of 2.4 years provides some reassurance about the relative stability of single time-point assessments of maltreatment in both research and applied settings, such as legal, social care and clinical contexts. This supports the use of retrospective measures as a stable feature, allowing researchers to categorize individuals as maltreated or non-maltreated with reasonable confidence, at least in the short term. It also suggests that the lack of agreement between prospective and retrospective measures is unlikely to be driven by shifting retrospective reports over time (that is, measurement error)^14^ and instead may be driven by stable individual differences in the way individuals do or do not recall maltreatment experiences^2,7^. More research is needed to better understand the developmental mechanisms underlying age-related differences in the stability of childhood maltreatment reports^10–13^.

There are nuanced implications for practice. The overall high stability of childhood maltreatment reports suggests that disclosures should be taken seriously and guide interventions in legal–forensic and social care settings. Nevertheless, a 20% change in reports highlights that professionals should anticipate some differences in reporting over time, particularly in children. In such cases, it is important to avoid premature conclusions about fabrication. Differences in reporting can reflect multiple processes, including changes in appraisal, the reconstructive nature of memory, changing motivational or contextual influences and errors^7^. This suggests that records of disclosure should include an account of the context and developmental factors. It also suggests the need to strengthen memory and developmental expertise among professionals working in this area. These implications for practice should be considered with caution, given the known research–practice gap: research and practice settings for reporting can be very different, and the greater stress, fear, stakes and lack of familiarity in practice settings might further influence report stability.

From a clinical perspective, our findings indicate that recall and cognitions surrounding maltreatment experiences are unlikely to change spontaneously over a few years, particularly in clinical populations. Retrospective recall of maltreatment is associated with heightened risk for subsequent psychiatric disorders, even accounting for previous history of psychopathology^87^. Therefore, the stability of maltreatment self-reports may help explain their association with chronicity and recurrence of emotional disorders^88,89^. Targeted interventions may be necessary to shift unhelpful appraisals and prevent or mitigate the lingering impact of maltreatment on psychopathology. Existing trauma-focused interventions (for example, trauma-focused cognitive behavioral therapy, eye movement desensitization and reprocessing, and imagery rescripting)^90,91^ already target maladaptive post-traumatic cognitions and memory processes in PTSD and might also be beneficial for the much broader set of trauma-related psychiatric disorders^92^. Because of the greater malleability of childhood maltreatment memories in childhood and adolescent years, such interventions may be more beneficial when delivered early in life. Children’s autobiographical memories are encoded with immature hippocampal systems and undergo inefficient consolidation, which may leave memories of early traumatic experiences comparatively unstable and modifiable. During childhood and adolescence—before these memories are fully integrated into cortical networks and narrative identity structures—therapeutic interventions could more easily influence how these memories are retrieved, elaborated and reconsolidated. As a result, trauma-focused treatments delivered early in life may have greater potential to shape how traumatic memories are ultimately organized and incorporated into the child’s developing autobiographical memory, thereby reducing long-term psychopathology.

## Methods

### Data sources

Following a predefined protocol registered on Prospero (identifier CRD42023414023), we conducted a systematic review and meta-analysis following the Preferred Reporting Items for Systematic Reviews and Meta-analysis (PRISMA) and Meta-analysis of Observational Studies in Epidemiology (MOOSE) reporting guidelines (Supplementary Information). We searched Embase, PsycInfo and MEDLINE for peer-reviewed studies that measured the stability of self-reports of child maltreatment, were written in English and were published from database inception until 1 October 2025. Search terms were: (child* maltreatment, child* abuse, child* neglect, child* trauma, child* adver*, early life stress, adverse childhood experiences, ACEs) and (intrarater, test–retest, reliability, stability, reproducibility, consistency, repeatability, intraclass correlation coefficient, ICC, kappa).

### Study selection

Titles and abstracts of all articles captured by the search were independently screened by one doctoral student (O.C.) and independent raters with undergraduate degree training who were blind to each other’s decisions (inter-rater agreement *κ* = 0.62). Full texts of potentially eligible studies were then screened independently following the same procedure (inter-rater agreement *κ* = 0.67). Inconsistencies were discussed and resolved with the senior author (A.D.). Peer-reviewed longitudinal studies with self-reports of child maltreatment assessed at least twice in the same individual participants were included. Deviating from the study’s pre-registration plan, we decided to include only studies with a time interval of 2 months or longer. This decision was guided by our interest in examining the stability of maltreatment memories over time rather than the short-term test–retest reliability of maltreatment measures; this resulted in the exclusion of *k* = 56 studies assessing the psychometric properties of maltreatment measures being developed.

Child maltreatment was defined as any of the following experiences between birth and age 18 years: physical abuse, sexual abuse, emotional abuse, physical neglect, emotional neglect, institutional neglect or deprivation, harsh physical discipline or corporal punishment, or broader measures of adversity that included any of the above forms of maltreatment.

### Data extraction

Data were extracted independently by one doctoral student (O.C.) and double entered by an independent rater. Inconsistencies were discussed and resolved in consensus meetings. Data extraction included stability estimate(s), exposure type(s), sample characteristics (population representative, convenience or clinical sampling), measure type (interview versus questionnaire), demographic factors (sex and age at baseline and follow-up assessment), time interval between assessments, attrition rate and study quality characteristics (population representativeness of the sample, attrition rate, whether the measures were validated and whether the study controlled for the effects of sociodemographic or clinical variables on the stability estimate). For clinical samples, we also extracted data on the type of psychopathology and the presence of an intervention between baseline and follow-up

For studies that measured stability at multiple time points, the longest time interval was selected by default. This was guided by our interest in examining memory-related processes, which are more likely to emerge and become more pronounced over longer periods. However, in cases where a shorter interval provided a greater number of effect sizes, the shorter interval was chosen.

### Statistical analysis

All analyses were conducted in R using the metafor package.

Studies reported different types of stability effect sizes, including Pearson and Spearman correlation coefficients, ICCs and Cohen’s *κ* coefficients. We initially harmonized all stability effect sizes onto a correlational scale using a calibration model to estimate tetrachoric correlations from *κ* statistics when necessary (Supplementary Methods and Supplementary Fig. 1). We then used Fisher’s *z*-transformation for the correlation effect sizes to stabilize the variance and achieve an approximately normal distribution, allowing us to calculate the variance using the *z*-transformed effect sizes and corresponding sample sizes with the escalc function in R. For clarity in reporting, the meta-analytic results were then inverse *z*-transformed and presented as correlations.

We estimated the overall meta-analytic effect size for the stability of self-reports of childhood maltreatment using a multilevel model (including random-sampling variance, within-study variance and between-study variance) to account for the non-independence of the data, as many studies contributed multiple effect sizes, for example across maltreatment types or subgroups (such as patients versus controls or male versus female participants). We used the *I*^2^ statistic to measure heterogeneity in the meta-analytical estimate. We then conducted sensitivity analyses testing for publication bias (an extension of the Egger’s test for multilevel meta-analysis models) and undue influence of cohorts, studies or effect sizes (leave-one-out analyses). A sensitivity analysis was also conducted to assess whether excluding studies where tetrachoric correlations were estimated using the calibration model affected the overall meta-analytic estimate (*k* = 11, *n* = 34).

We used meta-regressions to test whether stability was moderated by a set of a priori-defined factors, including type of maltreatment, type of sample (population-representative samples, non-representative convenience samples and clinical samples), type of measure (questionnaire or interview), sex distribution, age at assessment (baseline and follow-up), time interval between assessments, percentage loss to attrition and study quality. We also conducted a post hoc moderation analysis examining whether stability was moderated by effect size type (ICC, Pearson or Spearman’s correlation, or tetrachoric correlation).

As autobiographical memory undergoes major developmental changes from adolescence to adulthood, we conducted post hoc analyses using a binary age variable distinguishing studies with a mean baseline age of 18 years or younger (childhood and adolescence) from those with a mean baseline age over 18 years (adulthood) and tested moderation effects. We also fitted an interaction model between baseline age and follow-up interval to examine whether the effects of age on reporting stability varied by follow-up length, as developmental processes affecting memory may become more pronounced over longer intervals^12^. To assess the sensitivity of these findings to how developmental periods are defined, we repeated the moderation and interaction analyses using alternative cut-offs at ages 21 and 24, reflecting broader definitions of adolescence and emerging adulthood proposed in developmental frameworks^93^. Finally, we tested whether any observed effects of age on stability could be explained by newly occurring experiences in participants under 18 by restricting the analyses to studies explicitly specifying the same reporting period across time points.

Finally, within the subset of studies using a clinical sample, we tested whether the type of psychopathology moderated the effect size estimates and whether the presence of an intervention between baseline and follow-up influenced the association. To assess whether healthy volunteers influenced stability estimates in clinical studies, we also conducted a sensitivity analysis restricting sampling to studies that did not include healthy volunteers.

### Reporting summary

Further information on research design is available in the Nature Portfolio Reporting Summary linked to this article.

## Supplementary information

Supplementary Information Supplementary Methods, Figs. 1–12 and Tables 1–7.

Reporting Summary

Peer Review File

## Data Availability

The data extraction table is available via Code Ocean at 10.24433/CO.8392361.v1.

## Code availability

The script for the analysis is available via Code Ocean at 10.24433/CO.8392361.v1.
