# Supplementary material for: Stability of childhood maltreatment self-reports: a systematic review and meta-analysis
Source: Nat Ment Health. 2026 Jul 3;4(8):1315–28. doi: 10.1038/s44220-026-00677-7 (PMC13441976; doi:10.1038/s44220-026-00677-7)
Supplement: Supplementary file 1 [file 44220_2026_677_MOESM1_ESM.pdf]

nature mental health

Supplementary information https://doi.org/10.1038/s44220-026-00677-7

Stability of childhood maltreatment self-reports: a systematic review and meta-analysis

In the format provided by the authors and unedited

Table of Contents

Supplementary methods 3

Supplementary Table 1. Study quality assessment 5

Supplementary Table 2. Sensitivity analysis of moderation by baseline age defined by three cut off points 7

Supplementary Table 3. Interaction between baseline age and follow-up time interval for three age categorisations 8

Supplementary Table 4. Sensitivity analysis of moderation by baseline age (three categorisations), excluding Breton et al. (2022) & Park et al. (2018) 9

Supplementary Table 5. Sensitivity analysis of interaction of baseline age (three categorisations) and follow-up interval, excluding Breton et al. (2022) and Park et al. (2018) 10

Supplementary Table 6. Characteristics of clinical samples 11

Supplementary Table 7. Sensitivity analysis including only effect sizes derived from fully clinical groups 13

Supplementary Figure 1. Comparison of linear (blue) and quadratic (red) models for calibrating kappa estimates and tetrachoric correlations 14

Supplementary Figure 2. PRISMA Flow chart 15

Supplementary Figure 3. Funnel plot. 16

Supplementary Figure 4. Leave-one-out analysis, excluding each study in turn 17

Supplementary Figure 5. Leave-one-out analysis, excluding each cohort in turn. 18

Supplementary Figure 6. Bubble plot illustrating a linear model for the association between baseline ages and effect sizes from individual studies 19

Supplementary Figure 7. Logarithmic model for the association between baseline ages and effect sizes from individual studies 19

Supplementary Figure 8. Predictions from interaction model between baseline age (age 21 cut-off) and follow-up interval 20

Supplementary Figure 9. Predictions from interaction model between baseline age (age 24 cut-off) and follow-up interval 20

Supplementary Figure 10. Sensitivity analysis excluding Breton et al. (2022) and Park et al. (2018): predictions from the interaction model between baseline age (age 18 cut-off) and follow-up interval 21

Supplementary Figure 11. Sensitivity analysis excluding Breton et al. (2022) and Park et al. (2018): predictions from the interaction model between baseline age (age 21 cut-off) and follow-up interval 21

Supplementary Figure 12. Sensitivity analysis excluding Breton et al. (2022) and Park et al. (2018): predictions from the interaction model between baseline age (age 24 cut-off) and follow-up interval 22

Supplementary methods

Data harmonisation

Studies reported different types of stability effect sizes including Pearson's correlations, Spearman's correlations, intraclass correlation coefficients (ICCs), and Cohen's kappa coefficients. We harmonised all stability estimates onto a correlational scale, converting kappa coefficients to tetrachoric correlations. When studies reported both kappa coefficients and 2x2 contingency tables (k=15, n=55), we calculated tetrachoric correlations directly from the tables. We also used this subset of studies to develop a calibration model predicting tetrachoric correlations from kappa values and applied the model to derive tetrachoric correlations for studies that reported kappa coefficients but did not provide 2x2 tables (k=11, n=34). To develop this calibration model, we evaluated the performance of both linear and quadratic fits using leave-one-author-out cross-validation. In this process, studies from a single author were held out as a test set, while the remaining data were used for model training, looping across all authors. Both models performed well, but the quadratic model was superior: it had a lower average root mean squared error (7% deviation from the true value vs. 9% for the linear model) and a higher R^2 (93% vs. 87%) (**Supplementary Figure 1**). Therefore, the quadratic model was selected.

The decision to harmonise effect sizes onto the correlational scale was data-driven, as the majority of studies reported effect sizes in this form. Specifically, 63% of all effect sizes were reported as Pearson's, Spearman's, or intraclass correlation coefficients. An additional 23% were derived from 2×2 contingency tables, allowing tetrachoric correlations to be directly calculated. Only 14% of effect sizes required estimation of tetrachoric correlations from kappa values using the calibration model.

Data analysis

As most studies reported multiple effect sizes (e.g., for different maltreatment subtypes or subgroups, such as clinical participants vs. controls or male vs. female participants), we used multi-level random effects meta-analysis models to account for these dependencies. We initially specified four levels of variance in effect sizes: random-sampling variance, within-study variance, between-study variance, and between-sample variance. However, we eventually omitted the between-sample level as it did not capture any additional variance in effect sizes.

We estimated the overall meta-analytic effect size for the stability of self-reports of childhood maltreatment. To estimate heterogeneity, we used the I^2 statistic, which reflects the proportion of the observed variance that is due to true variation in effect sizes if sampling error was eliminated.

We then conducted sensitivity analyses testing for publication bias and undue influence of cohorts, studies, or effect sizes. To test for publication bias, we used an extension of the Egger's test for multilevel meta-analysis models, which tests whether study variance moderates the meta-analytic effect size. We conducted three leave-one-out

analyses to test for the undue influence of individual studies by examining changes in estimates across permutations, which omitted each cohort, study, or effect size in turn. A sensitivity analysis was also conducted to assess whether excluding studies where tetrachoric correlations were estimated using the calibration model affected the overall meta-analytic estimate.

We based our qualitative description of the correlational effect sizes on conventions suggestion by Cohen, which identifies $r>.5$ as “large” (Cohen, 1988).

Supplementary Table 1. Study quality assessment. Each study was scored on four criteria: (i) sample representativeness (1 = population representative; 0 = not population representative), (ii) (1 = <60% loss; 0 = ≥60% loss), (iii) measure validation (1 = validated measure; 0 = non-validated), and (iv) whether the study tested for moderation by sociodemographic or clinical factors (1 = tested; 0 = not tested). Scores were summed to generate a total quality score ranging from 0 to 4.

**Source**	**Sample representativeness**	**Loss to attrition**	**Validated measure**	**Moderation**	**Total**
Aalsma et al., 2002	0	1	0	1	2
Bernstein et al., 1994	0	0	0	0	0
Bifulco et al., 2005	0	0	1	0	1
Boisselier et al., 2023	0	0	1	0	1
Breton et al., 2022	1	1	1	1	4
Cammack et al., 2016	0	0	1	0	1
Cay et al., 2022	0	1	1	1	3
Chatziioannidis et al., 2021	0	1	1	0	2
Colman et al., 2016	1	1	0	1	3
Dube et al., 2004	0	0	1	1	2
Durrett et al., 2004	0	1	1	1	3
Fergusson et al., 2011	1	1	0	1	3
Fisher et al., 2011	0	0	1	0	1
Frampton et al., 2018	0	1	1	1	3
Friedrich et al., 1997	1	1	1	1	4
Goldberg et al., 2006	0	1	0	0	1
Goldsmith et al., 2009	0	1	0	1	2
Goltermann et al., 2023	0	1	1	1	3
Hagborg et al., 2022	1	1	1	0	3
Hardt et al., 2006	0	1	1	0	2
Hoeboer et al., 2025	0	1	1	0	2
Horberg et al., 2019	0	0	1	0	1
Hosang et al., 2023	0	1	1	1	3

Kerbage et al., 2025	0	0	1	1	2
Kremers et al., 2007	0	1	0	1	2
McKinney et al., 2009	1	1	0	1	3
Mersky et al., 2017	0	0	1	0	1
Mesquita et al., 2018	0	0	1	1	2
Naicker et al., 2017	1	1	0	0	2
Nelson et al., 2010	0	0	0	0	0
Okeke et al., 2017	1	1	0	1	3
Paivio et al., 2001	0	1	1	0	2
Paivio et al., 2004	0	0	1	0	1
Park et al., 2018	0	1	1	0	2
Peng et al., 2023	1	0	1	0	2
Pereira da Silva et al., 2013	0	1	1	0	2
Pinto et al., 2014	0	1	1	1	3
Plaza et al., 2011	0	0	1	0	1
Racine et al., 2020	0	1	0	1	2
Shannon et al., 2016	0	1	1	0	2
Simpson et al., 2019	0	1	1	1	3
Smith et al., 2002	0	0	1	0	1
Spinhoven et al., 2012	0	1	1	1	3
Teicher et al., 2015	0	0	1	0	1
Wielaard et al. 2018	0	1	1	1	3
Xiang et al., 2021	0	1	1	0	2
Yancura et al., 2009	0	1	1	1	3
Zanotti et al., 2018	0	1	1	0	2
Zhang et al., 2024	0	1	1	0	2

Supplementary Table 2. Sensitivity analysis of moderation by baseline age defined by three cut off points (at age 18, age 21, and age 24)

Moderator	k	N	No. ES	r (95% CI)	Q_mod	p-value	% variance
Age 18 cut-off					8.88	0.009	7.63
0-18	8	5847	32	0.65 (0.49, 0.77)			
19+	40	25019	202	0.81 (0.77, 0.85)			
Age 21 cut-off					10.39	0.001	10.40
0-21	15	7536	57	0.68 (0.57, 0.79)			
21+	34	23354	177	0.83 (0.79, 0.86)			
Age 24 cut-off					7.98	0.005	8.56
0-24	18	20097	69	0.71 (0.62, 0.78)			
25+	30	10769	165	0.83 (0.79, 0.86)			

Supplementary Table 3. Interaction between baseline age and follow-up time interval for three age categorisations. Note: estimates reported on z-transformed scale

	Z-transformed effect sizes (95% CI)	P-value	Q_mod	p-value	% variance
**Age 18 cut-off**			15.81	0.0012	15.40
Intercept	1.03 (0.73, 1.33)	<0.0001			
Baseline age (binary) (0 = 0-18, 1= 19+)	0.16 (-0.17, 0.49)	0.36			
Follow up time interval	-0.003 (-0.01, -0.001)	0.01			
Interaction	0.002 (0.0001, 0.005)	0.04			
**Age 21 cut-off**			25.20	<0.0001	22.66
Intercept	1.08 (0.87, 1.30)	<0.0001			
Baseline age (binary) (0 = 0-21, 1= 22+)	0.16 (-0.10, 0.41)	0.24			
Follow up time interval	-0.003 (-0.005, -0.001)	0.001			
Interaction	0.003 (0.001, 0.005)	0.01			
**Age 24 cut-off**			22.90	<0.0001	21.31
Intercept	1.11 (0.92, 1.31)	<0.0001			
Baseline age (binary) (0 = 0-24, 1= 25+)	0.10 (-0.15, 0.35)	0.42			
Follow up time interval	-0.003 (-0.004, -0.001)	0.0003			
Interaction	0.002 (0.001, 0.004)	0.01			

Supplementary Table 4. Sensitivity analysis of moderation by baseline age (three categorisations), excluding Breton et al. (2022) & Park et al. (2018)

Moderator	k	N	No. ES	r (95% CI)	Q_mod	p-value	% variance
Age 18 cut-off					2.66	0.10	11.13
0-18	6	3918	25	0.71 (0.55, 0.82)			
19+	40	25019	202	0.81 (0.77, 0.85)			
Age 21 cut-off					6.65	0.01	7.70
0-21	13	5607	50	0.72 (0.61, 0.79)			
22+	34	23354	177	0.83 (0.79, 0.86)			
Age 24 cut-off					5.20	0.02	14.00
0-24	16	18168	62	0.74 (0.65, 0.80)			
25+	30	10769	165	0.83 (0.79, 0.86)			

Supplementary Table 5. Sensitivity analysis of interaction of baseline age (three categorisations) and follow-up interval, excluding Breton et al. (2022) and Park et al. (2018). Note: on the z-transformed scale.

	Z-transformed effect sizes (95% CI)	P-value	Q_mod	p-value	% variance
**Age 18 cut-off**			8.90	0.03	16.18
Intercept	1.11 (0.79, 1.43)	<0.0001			
Baseline age (binary) (0 = 0-18, 1= 19+)	0.07 (-0.28, 0.43)	0.69			
Follow up time interval	-0.002 (-0.01, -0.0003)	0.03			
Interaction	0.002 (-0.0003, 0.004)	0.09			
**Age 21 cut-off**			17.4	0.0006	23.56
Intercept	1.12 (0.89, 1.34)	<0.0001			
Baseline age (binary) (0 = 0-21, 1= 22+)	0.12 (-0.14, 0.38)	0.37			
Follow up time interval	-0.003 (-0.005, -0.0009)	0.003			
Interaction	0.002 (0.0003, 0.004)	0.03			
**Age 24 cut-off**			17.1	<0.001	23.80
Intercept	1.15 (0.96, 1.34)	<0.0001			
Baseline age (binary) (0 = 0-24, 1= 25+)	0.07 (-0.18, 0.31)	0.61			
Follow up time interval	-0.002 (-0.004, -0.001)	0.001			
Interaction	0.002 (0.0005, 0.004)	0.01			

Supplementary Table 6. Characteristics of clinical samples

**Author**	**Diagnosis (coded)**	**Proportion diagnosis (%)**	**Intervention**
Bernstein et al.,1994	Drug or alcohol addiction (addiction)	100	Undergoing treatment for drug and/or alcohol dependence including detoxification, rehabilitation, outpatient methadone maintenance and 'drug-free' groups
Bifulco et al., 2005	Depression (depression)	38	
Cammack et al., 2016	Mental health problems (general)	100	
Cay et al., 2022	Schizophrenia spectrum disorder or bipolar I disorder with psychotic features (Psychosis, Bipolar)	0, 56,100 (reliability reported separately for patients and controls, as well as for the entire sample)	
Chatziioannidis et al., 2021	Schizophrenia and other psychotic disorders (psychosis)	100	
Durrett et al., 2004	Borderline personality disorder features (personality disorder)	43	
Goltermann et al., 2019	Depression (depression)	45-53	Inpatient treatment for a current depressive episode for one cohort
Hoeboer et al., 2025	PTSD (PTSD)	100	Participants were randomised to one of three parallel conditions: Prolonged Exposure, intensified Prolonged Exposure, and Skills Training in Affective and Interpersonal Regulation followed by Prolonged Exposure
Horberg et al., 2019	BPD, bipolar disorder, ADHD (general)	100	
Hosang et al., 2023	Bipolar disorder (bipolar)	100	
Kerbage et al., 2025	Depression (depression)	76	
Kremers et al., 2007	Borderline personality disorder (personality disorder)	100	27 months of intensive treatment with schema focused therapy or transference focused psychotherapy
Mesquita et al., 2018	DSM-IV diagnosis (general)	100	
Paivio et al., 2001	Range of diagnoses (general)	100	20 sessions of emotion focused therapy incorporating techniques similar to those used in process experiential therapy and

			principals similar to exposure with an emphasis on constructing new meaning
Park et al., 2018	Depression (depression)	46.5	
Shannon et al., 2016	Bipolar disorder	100	
Simpson et al., 2019	First episode psychosis (psychosis)	0, 100 (reliability reported separately for patients & controls)	
Smith et al., 2002	Range of diagnoses (general)	100	
Spinhoven et al., 2012	Personality disorder (personality disorder)	100	12 months of long-term treatment of personality disorders - patients randomly assigned to schema-focused therapy, clarification oriented psychotherapy or treatment as usual: the proportion of patients with increasing or decreasing trauma reports did not differ between treatment conditions
Wielaard et al., 2018	Depression (depression)	65	
Xiang et al., 2021	Schizophrenia (psychosis)	100	

Supplementary Table 7. Sensitivity analysis including only effect sizes derived from fully clinical groups (excluding healthy volunteers), based on studies exclusively recruiting clinical samples or studies reporting separate estimates for clinical vs control groups; testing moderation by diagnosis and intervention.

**Moderator**	**k**	**N**	**No. ES**	**r (95% CI)**	**Q_mod**	**p-value**
Diagnosis					1.03	0.96
Addiction	1	40	6	0.83 (0.56, 0.94)		
Bipolar	3	109	17	0.81 (0.68, 0.89)		
General psychopathology	5	383	22	0.82 (0.72, 0.89)		
Personality disorder	2	229	6	0.88 (0.73, 0.94)		
Psychosis	5	154	24	0.80 (0.70, 0.88)		
PTSD	1	100	5	0.83 (0.56, 0.94)		
Intervention					0.57	0.45
No intervention	5	402	59	0.81 (0.75, 0.86)		
Intervention	11	596	21	0.84 (0.77, 0.90)		

Supplementary Figure 1. Comparison of linear (blue) and quadratic (red) models for calibrating kappa estimates and tetrachoric correlations, based on n=55 effect sizes from k=15 studies.

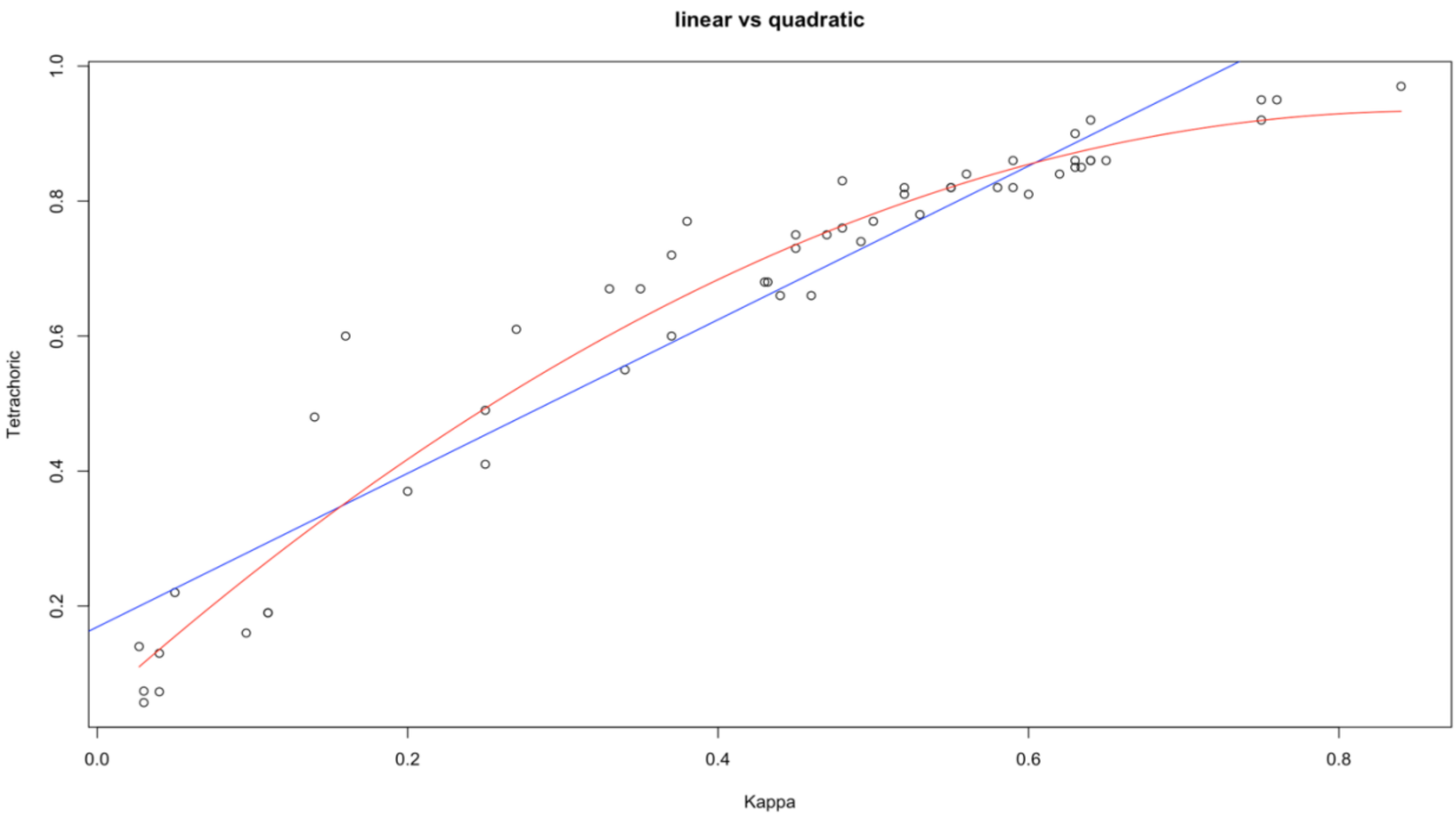

Supplementary Figure 2. PRISMA Flow chart

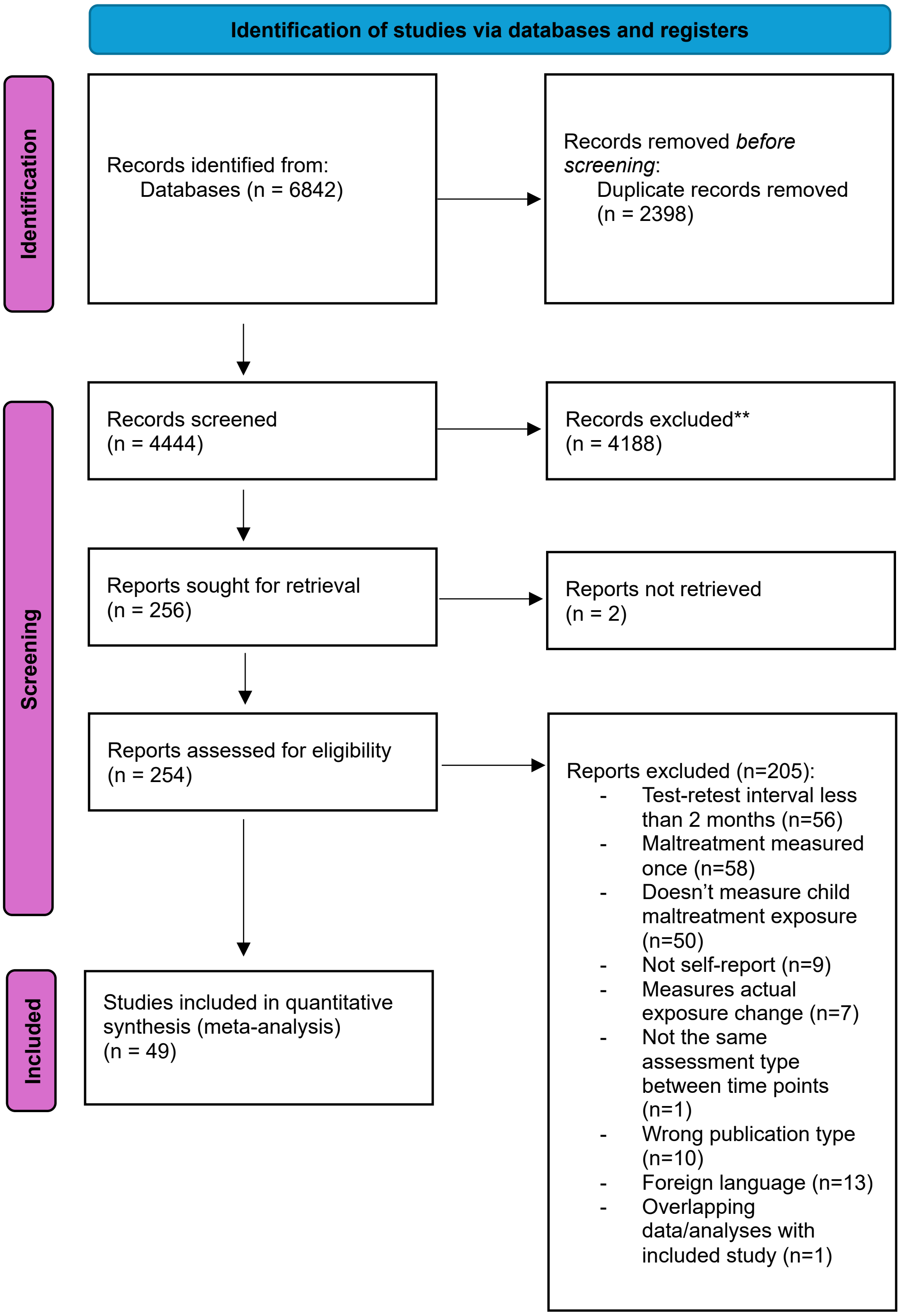

Supplementary Figure 3. Funnel plot. Colours represent individual studies

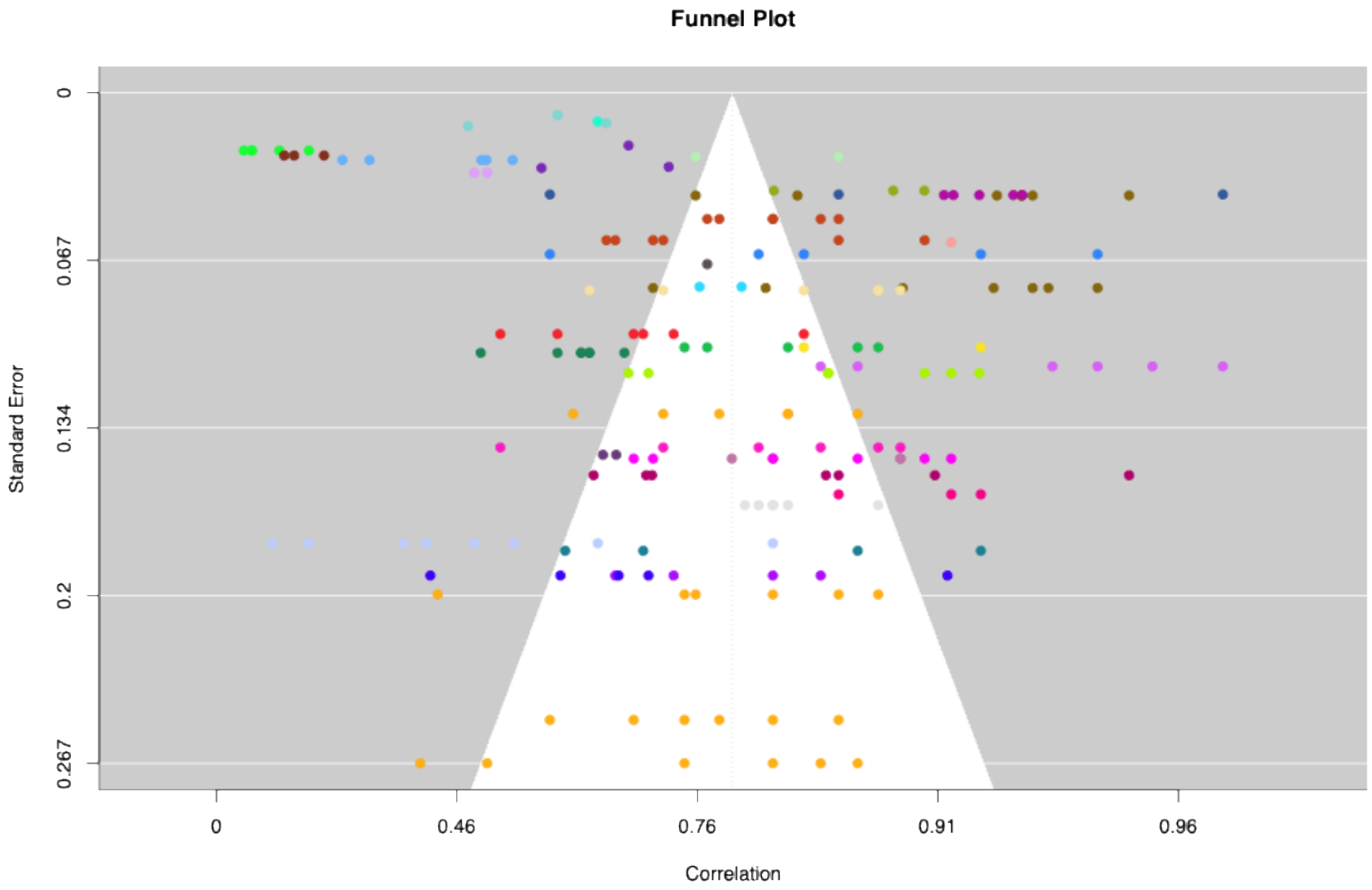

Supplementary Figure 4. Leave-one-out analysis, excluding each study in turn

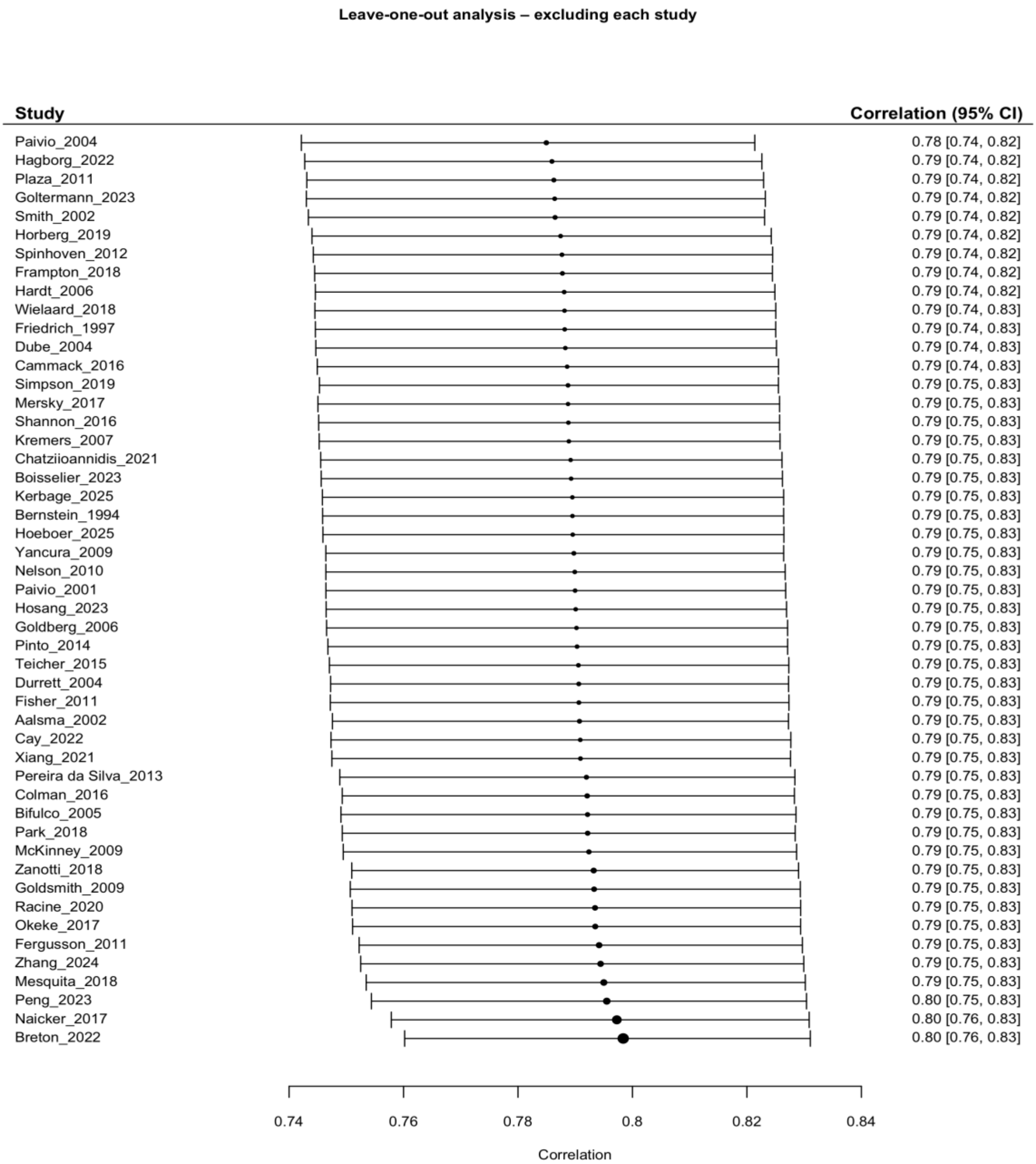

Supplementary Figure 5. Leave-one-out analysis, excluding each cohort in turn.

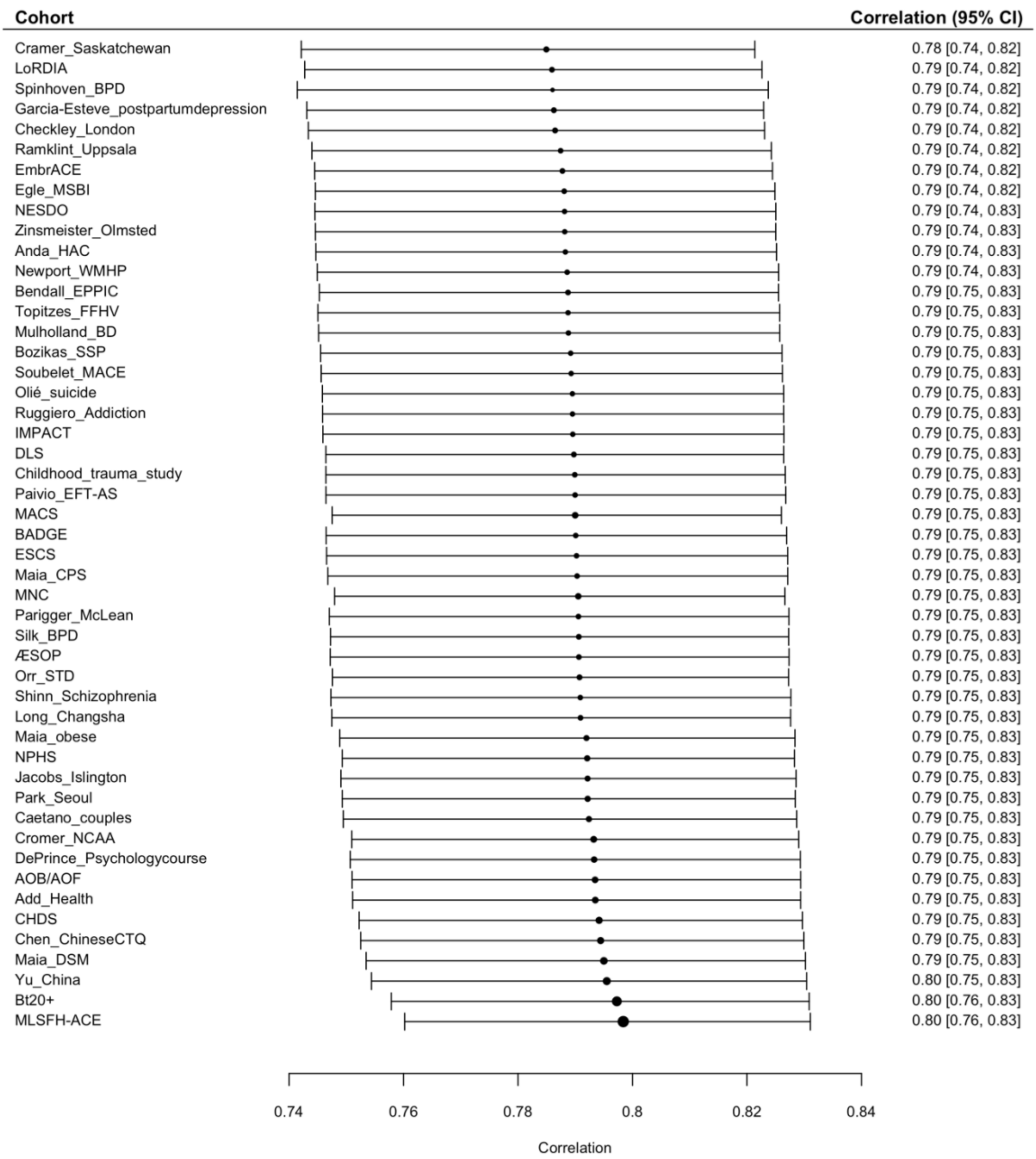

Supplementary Figure 6. Bubble plot illustrating a linear model for the association between baseline ages and effect sizes from individual studies (the bubble size reflects the meta-analytic weighting of each effect size).

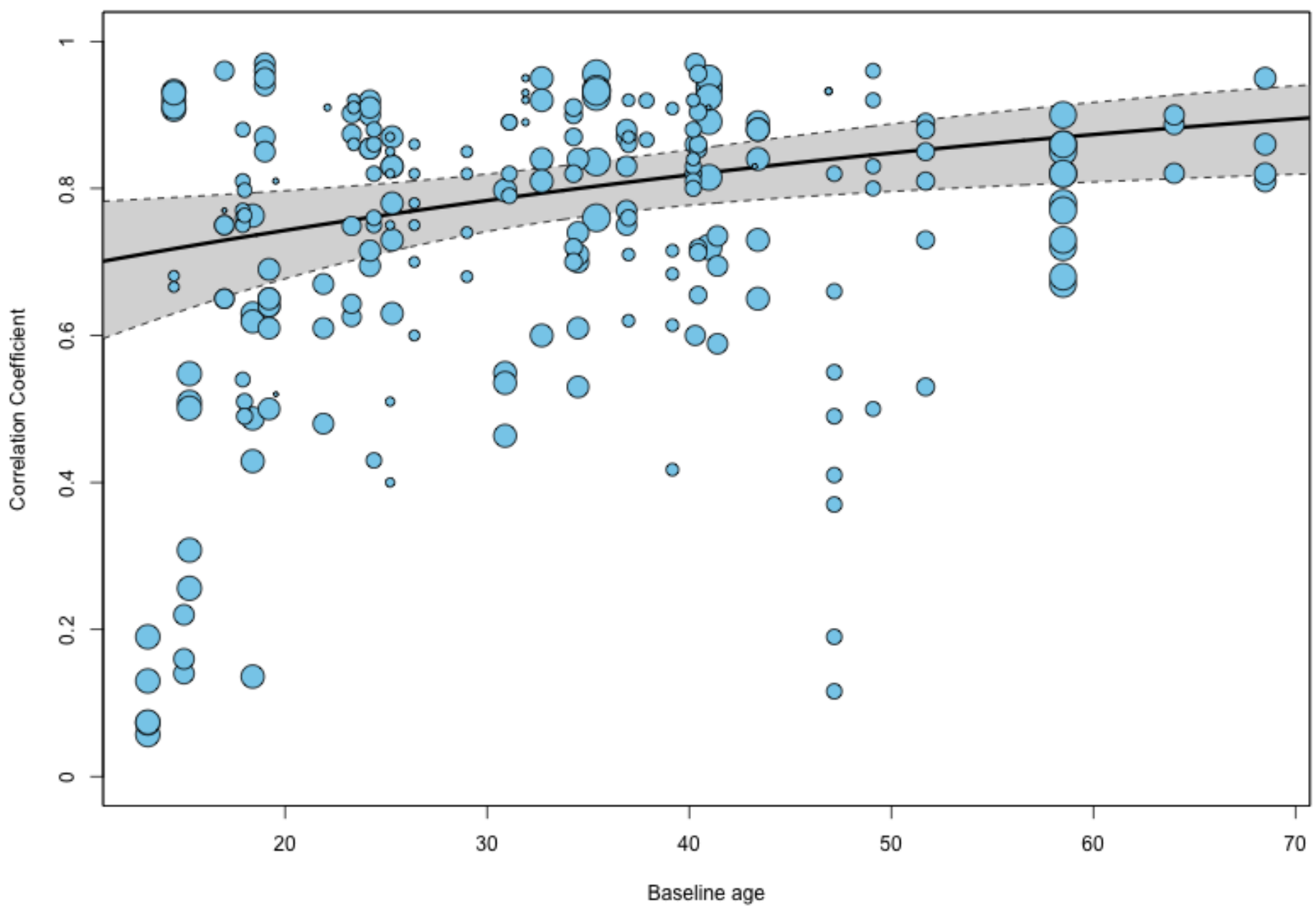

Supplementary Figure 7. Logarithmic model for the association between baseline ages and effect sizes from individual studies

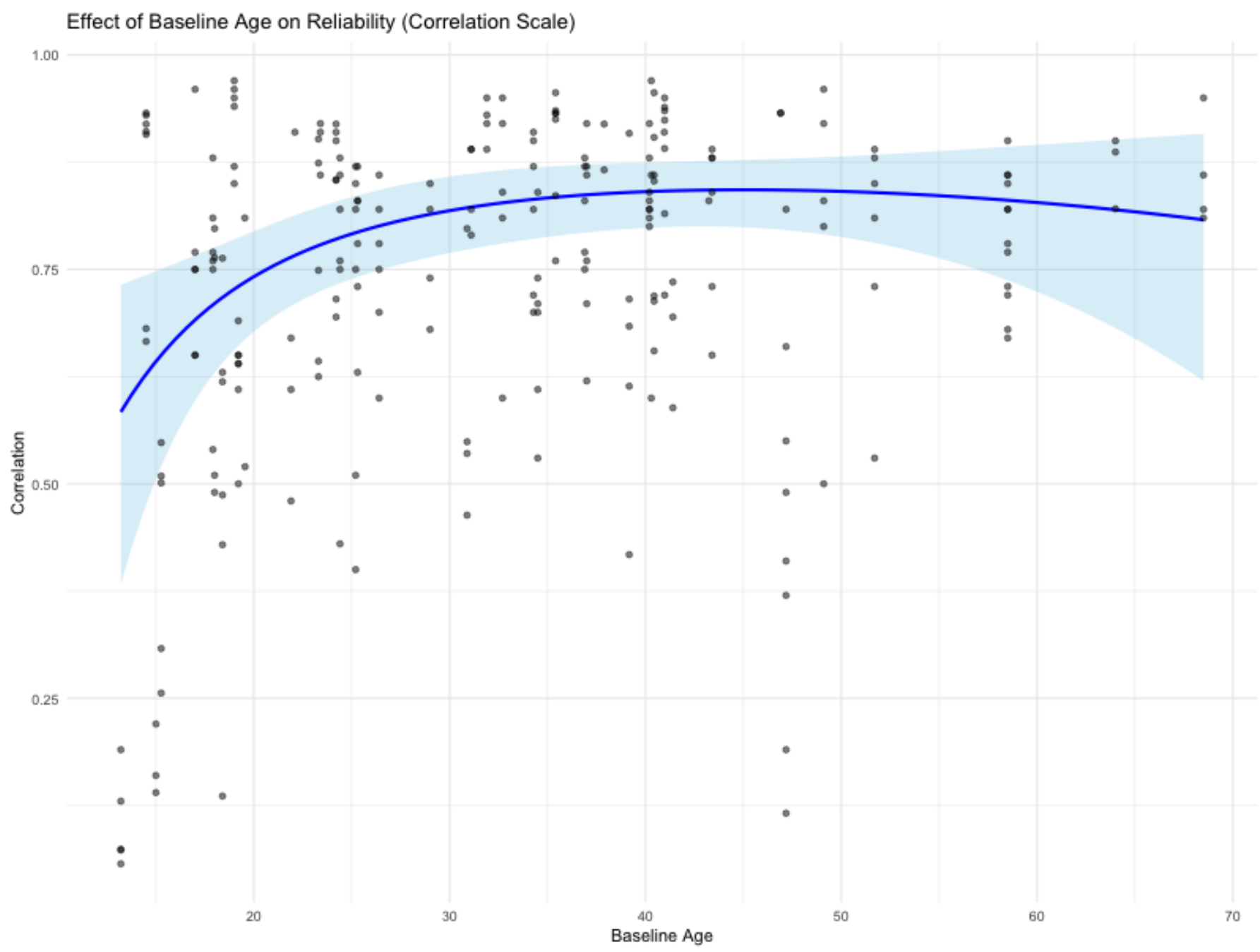

Supplementary Figure 8. Predictions from interaction model between baseline age (age 21 cut-off) and follow-up interval, inverse transformed onto the correlation scale for 0-261 weeks (0-5 years)

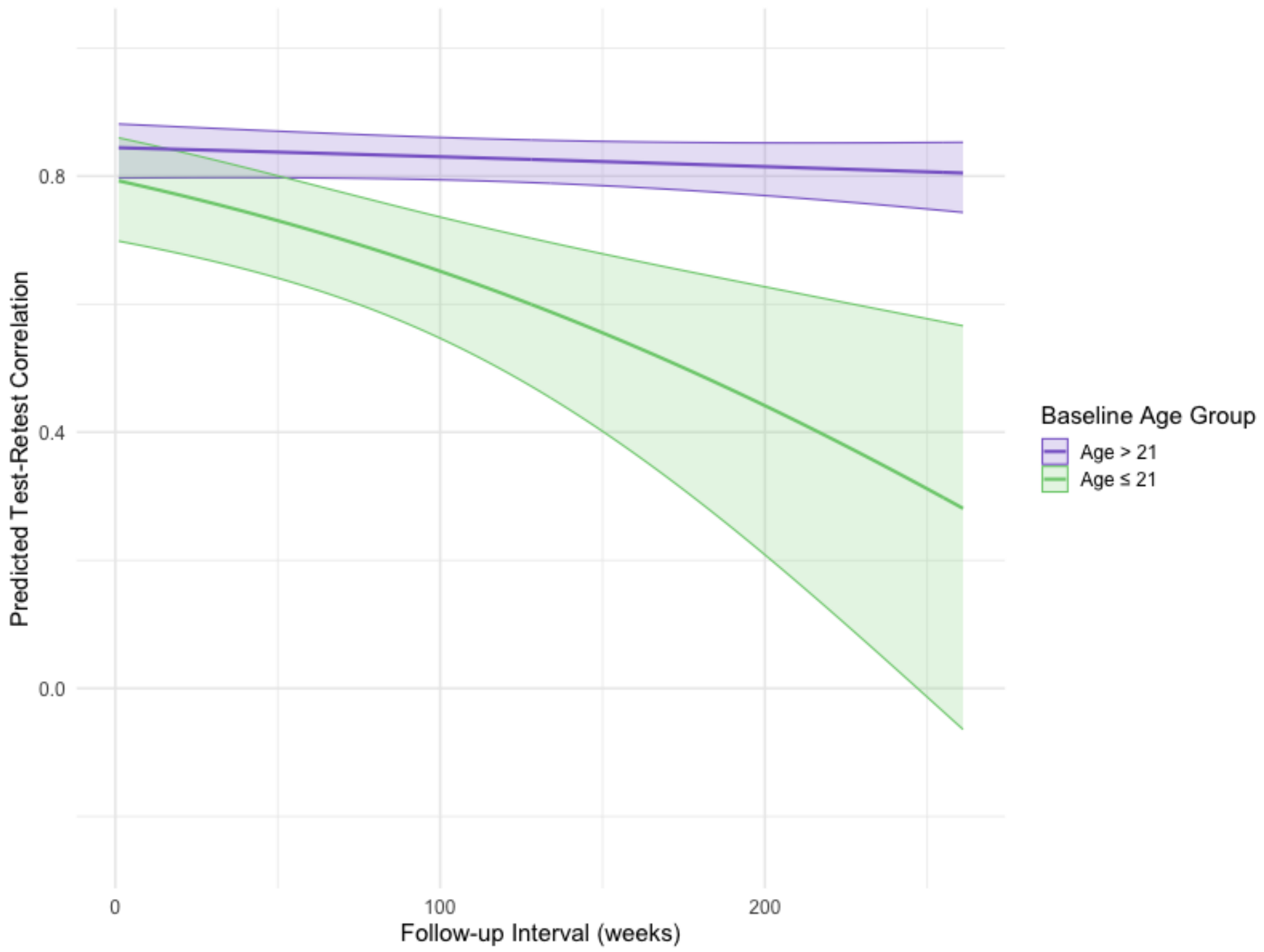

Supplementary Figure 9. Predictions from interaction model between baseline age (age 24 cut-off) and follow-up interval, inverse transformed onto the correlation scale for 0-261 weeks (0-5 years)

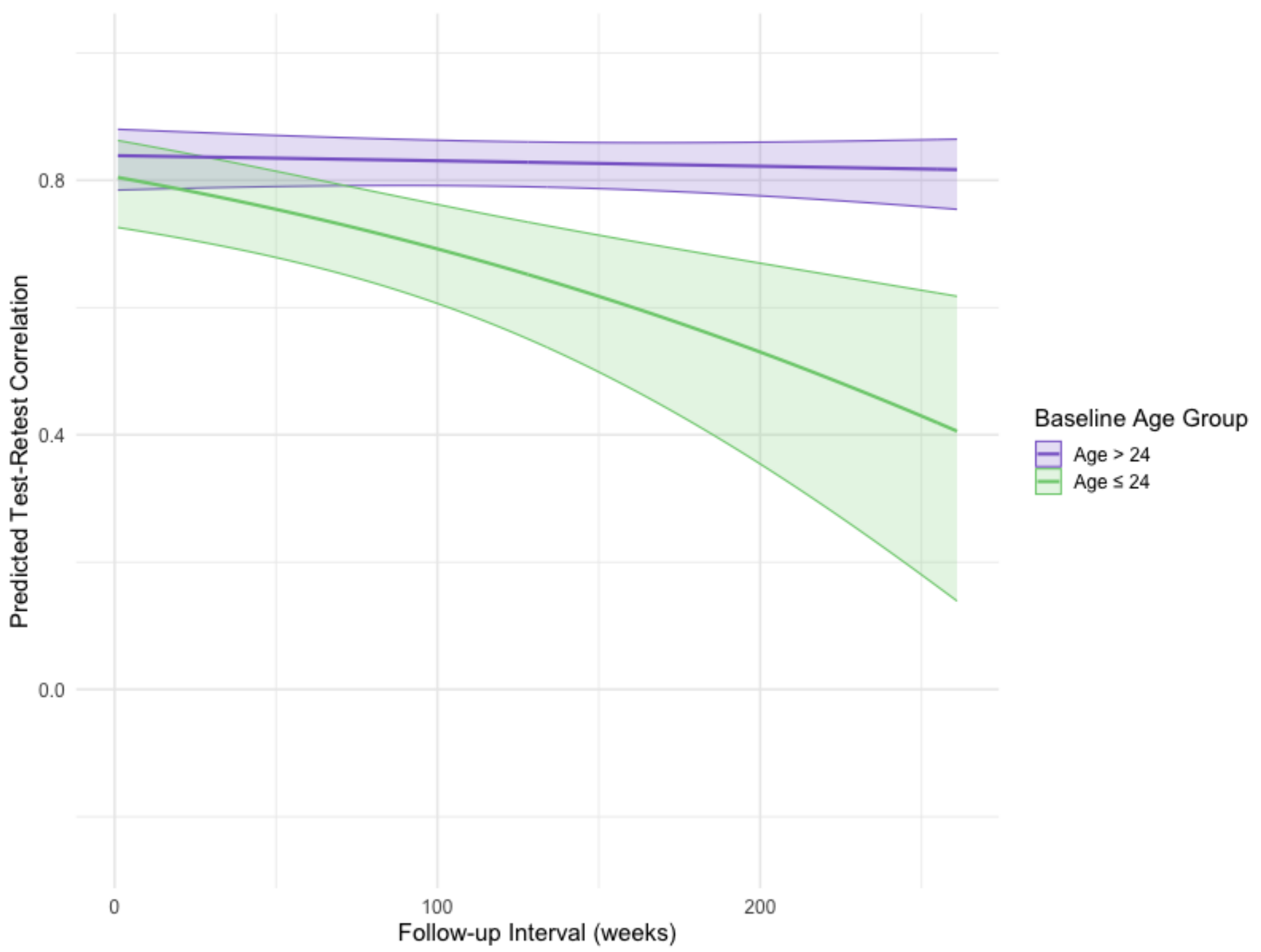

Supplementary Figure 10. Sensitivity analysis excluding Breton et al. (2022) and Park et al. (2018): predictions from the interaction model between baseline age (age 18 cut-off) and follow-up interval, inverse-transformed onto the correlation scale for 0-261 weeks

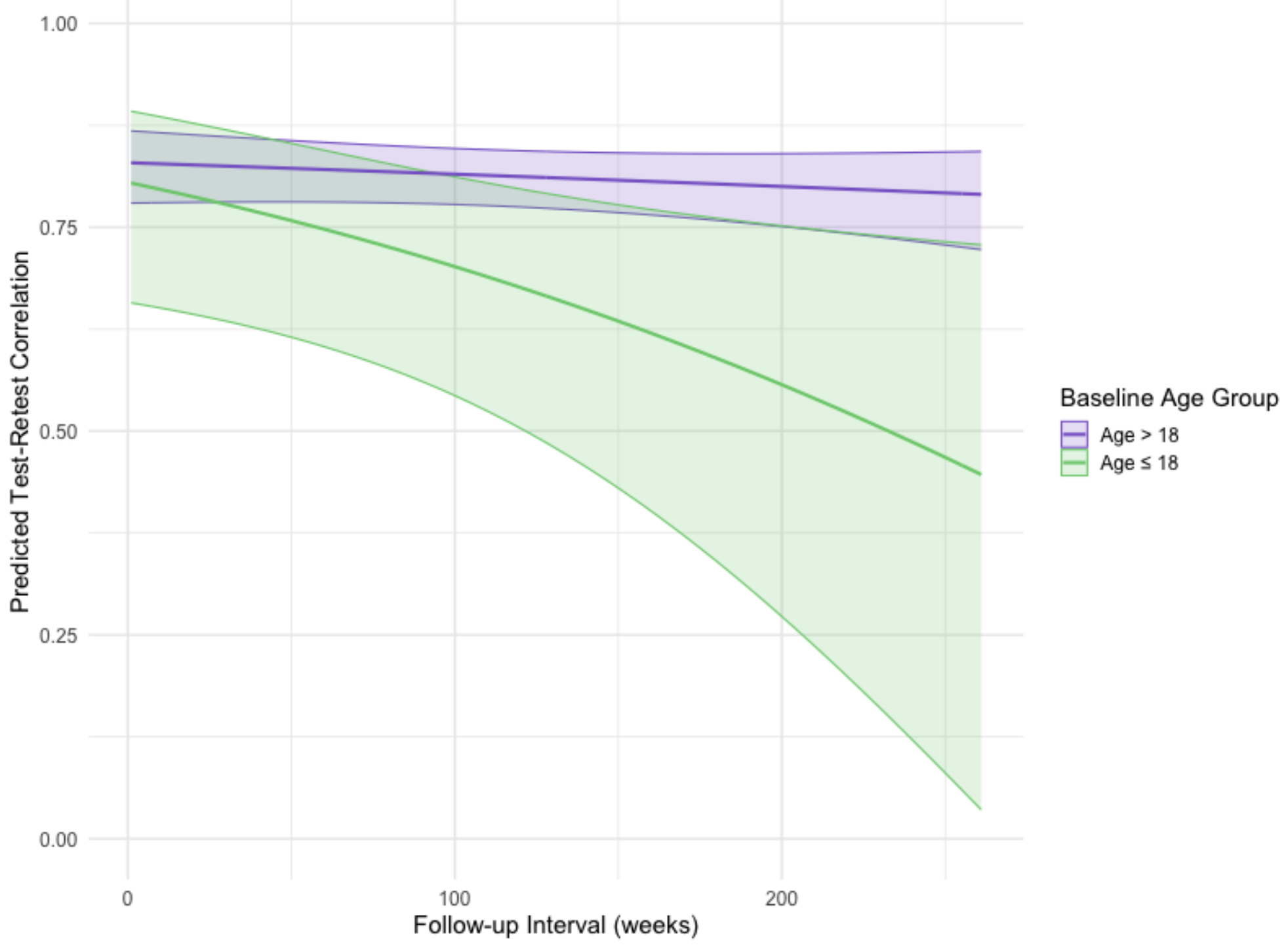

Supplementary Figure 11. Sensitivity analysis excluding Breton et al. (2022) and Park et al. (2018): predictions from the interaction model between baseline age (age 21 cut-off) and follow-up interval, inverse-transformed onto the correlation scale for 0-261 weeks

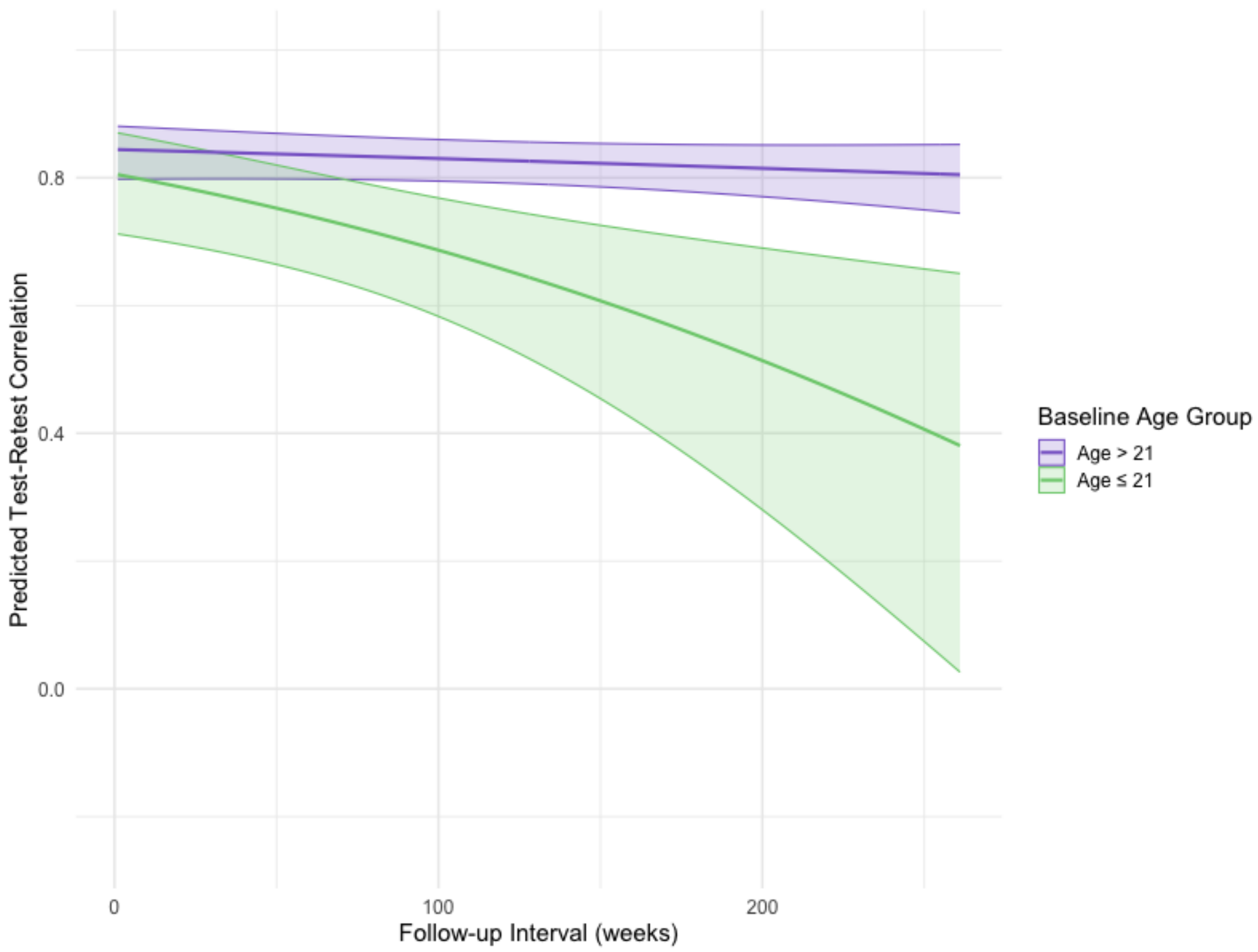

Supplementary Figure 12. Sensitivity analysis excluding Breton et al. (2022) and Park et al. (2018): predictions from the interaction model between baseline age (age 24 cut-off) and follow-up interval, inverse-transformed onto the correlation scale for 0-261 weeks

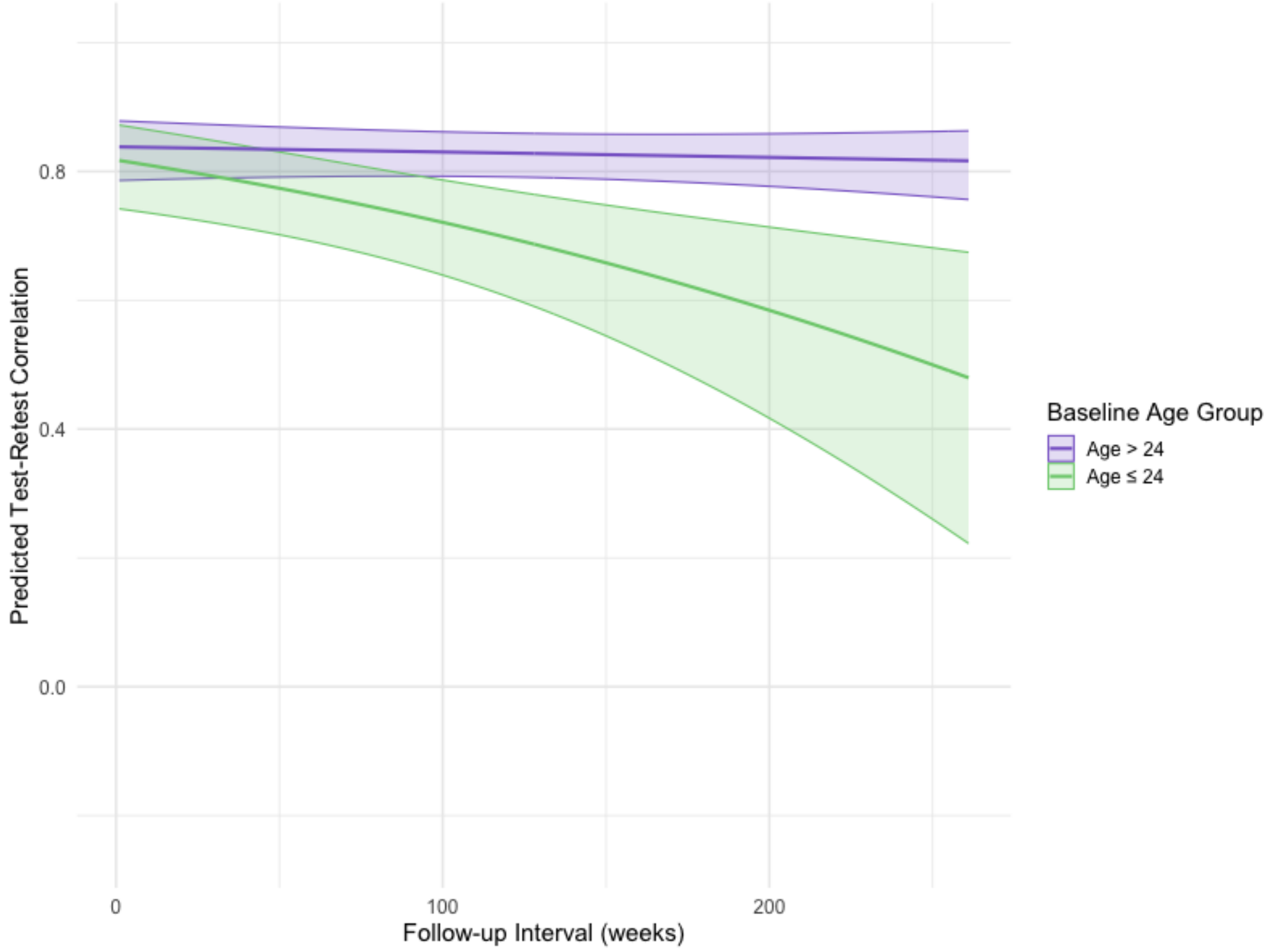

References

Aalsma, M. C., Zimet, G. D., Fortenberry, J. D., Blythe, M., & Orr, D. P. (2002). Reports of childhood sexual abuse by adolescents and young adults: Stability over time. *The Journal of Sex Research*, *39*(4), 259–263. https://doi.org/10.1080/00224490209552149

Bernstein, D. P., Fink, L., Handelsman, L., Foote, J., Lovejoy, M., Wenzel, K., Sapareto, E., & Ruggiero. (1994). Initial reliability and validity of a new retrospective measure of child abuse and neglect. *American Journal of Psychiatry*, *151*(8), 1132–1136. https://doi.org/10.1176/ajp.151.8.1132

Bifulco, A., Bernazzani, O., Moran, P. M., & Jacobs, C. (2005). The childhood experience of care and abuse questionnaire (CECA.Q): Validation in a community series. *British Journal of Clinical Psychology*, *44*(4), 563–581. https://doi.org/10.1348/014466505X35344

Boisselier, N., & Soubelet, A. (2023). Measuring early life adversity in adults: French translation and validation of the Maltreatment and Abuse Chronology of Exposure (MACE) scale. *European Journal of Trauma & Dissociation*, *7*(3), 100336. https://doi.org/10.1016/j.ejtd.2023.100336

Breton, E., Kidman, R., Behrman, J., Mwera, J., & Kohler, H.-P. (2022). Longitudinal consistency of self-reports of adverse childhood experiences among adolescents in a low-income setting. *SSM - Population Health*, *19*, 101205. https://doi.org/10.1016/j.ssmph.2022.101205

Cammack, A. L., Hogue, C. J., Drews-Botsch, C. D., Kramer, M. R., Pearce, B. D., T. Knight, B., Stowe, Z. N., & Newport, D. J. (2016). Test-retest reliability of retrospective self-reported maternal exposure to childhood abuse and neglect.

Archives of Women's Mental Health, *19*(2), 415–421. https://doi.org/10.1007/s00737-015-0536-x

Cay, M., Chouinard, V.-A., Hall, M.-H., & Shinn, A. K. (2022). Test-retest reliability of the Childhood Trauma Questionnaire in psychotic disorders. *Journal of Psychiatric Research*, *156*, 78–83. https://doi.org/10.1016/j.jpsychires.2022.09.053

Chatziioannidis, S., Agorastos, A., Kaprinis, S., & Bozikas, V.-P. (2021). Short-term reliability of retrospective childhood trauma reports in schizophrenia spectrum psychosis. *Psychiatriki*. https://doi.org/10.22365/jpsych.2021.024

Cohen, J. (1988). *Statistical Power Analysis for the Behavioural Sciences*. Routledge.

Colman, I., Kingsbury, M., Garad, Y., Zeng, Y., Naicker, K., Patten, S., Jones, P. B., Wild, T. C., & Thompson, A. H. (2016). Consistency in adult reporting of adverse childhood experiences. *Psychological Medicine*, *46*(3), 543–549. https://doi.org/10.1017/S0033291715002032

Dube, S. R., Williamson, D. F., Thompson, T., Felitti, V. J., & Anda, R. F. (2004). Assessing the reliability of retrospective reports of adverse childhood experiences among adult HMO members attending a primary care clinic. *Child Abuse & Neglect*, *28*(7), 729–737. https://doi.org/10.1016/j.chiabu.2003.08.009

Durrett, C., Trull, T. J., & Silk, K. (2004). Retrospective Measures of Childhood Abuse: Concurrent Validity and Reliability in a Nonclinical Sample with Borderline Features. *Journal of Personality Disorders*, *18*(2), 178–192. https://doi.org/10.1521/pedi.18.2.178.32778

Fergusson, D. M., Horwood, L. J., & Boden, J. M. (2011). Structural equation modeling of repeated retrospective reports of childhood maltreatment: Modeling of reports

of child maltreatment. *International Journal of Methods in Psychiatric Research*, *20*(2), 93–104. https://doi.org/10.1002/mpr.337

Fisher, H. L., Craig, T. K., Fearon, P., Morgan, K., Dazzan, P., Lappin, J., Hutchinson, G., Doody, G. A., Jones, P. B., McGuffin, P., Murray, R. M., Leff, J., & Morgan, C. (2011). Reliability and Comparability of Psychosis Patients' Retrospective Reports of Childhood Abuse. *Schizophrenia Bulletin*, *37*(3), 546–553. https://doi.org/10.1093/schbul/sbp103

Frampton, N. M. A., Poole, J. C., Dobson, K. S., & Pusch, D. (2018). The effects of adult depression on the recollection of adverse childhood experiences. *Child Abuse & Neglect*, *86*, 45–54. https://doi.org/10.1016/j.chiabu.2018.09.006

Friedrich, W. N., Talley, N. J., Panser, L., Fett, S., & Zinmeister, A. R. (1997). Concordance of Reports of Childhood Abuse by Adults. *Child Maltreatment*, *2*, 164–171.

Goldberg, L. R., & Freyd, J. J. (2006). Self-Reports of Potentially Traumatic Experiences in an Adult Community Sample: Gender Differences and Test-Retest Stabilities of the Items in a Brief Betrayal-Trauma Survey. *Journal of Trauma & Dissociation*, *7*(3), 39–63. https://doi.org/10.1300/J229v07n03_04

Goldsmith, R. E., Freyd, J. J., & DePrince, A. P. (2009). To Add Insight to Injury: Childhood Abuse, Abuse Perceptions, and the Emotional and Physical Health of Young Adults. *Journal of Aggression, Maltreatment & Trauma*, *18*(4), 350–366. https://doi.org/10.1080/10926770902901527

Goltermann, J., Meinert, S., Hülsmann, C., Dohm, K., Grotegerd, D., Redlich, R., Waltemate, L., Lemke, H., Thiel, K., Mehler, D. M. A., Enneking, V., Borgers, T., Repple, J., Gruber, M., Winter, N., Hahn, T., Brosch, K., Meller, T., Ringwald, K. G., … Dannlowski, U. (2023). Temporal stability and state-dependence of

retrospective self-reports of childhood maltreatment in healthy and depressed adults. *Psychological Assessment*, *35*(1), 12–22. https://doi.org/10.1037/pas0001175

Hagborg, J. M., Kalin, T., & Gerdner, A. (2022). The Childhood Trauma Questionnaire—Short Form (CTQ-SF) used with adolescents – methodological report from clinical and community samples. *Journal of Child & Adolescent Trauma*, *15*(4), 1199–1213. https://doi.org/10.1007/s40653-022-00443-8

Hardt, J., Sidor, A., Bracko, M., & Egle, U. T. (2006). Reliability of Retrospective Assessments of Childhood Experiences in Germany. *Journal of Nervous & Mental Disease*, *194*(9), 676–683. https://doi.org/10.1097/01.nmd.0000235789.79491.1b

Hoeboer, C. M., Bodor, N., Oprel, D. A. C., De Kleine, R. A., Schoorl, M., Van Minnen, A., & Van Der Does, W. (2025). Validation of the Childhood Trauma Questionnaire (CTQ) in the Context of Trauma-Focused Treatment. *Child Maltreatment*, 10775595251328611. https://doi.org/10.1177/10775595251328611

Hörberg, N., Kouros, I., Ekselius, L., Cunningham, J., Willebrand, M., & Ramklint, M. (2019). Early Trauma Inventory Self-Report Short Form (ETISR-SF): Validation of the Swedish translation in clinical and non-clinical samples. *Nordic Journal of Psychiatry*, *73*(2), 81–89. https://doi.org/10.1080/08039488.2018.1498127

Hosang, G. M., Manoli, A., Shakoor, S., Fisher, H. L., & Parker, C. (2023). Reliability and convergent validity of retrospective reports of childhood maltreatment by individuals with bipolar disorder. *Psychiatry Research*, *321*, 115105. https://doi.org/10.1016/j.psychres.2023.115105

Kerbage, H., Picot, M. C., Charpentier, S., Leboyer, M., Courtet, P., & Olié, E. (2025). Influence of depressive symptoms and current suicidality on the stability of responses to the childhood trauma questionnaire. *Child Abuse & Neglect*, *167*, 107560. https://doi.org/10.1016/j.chiabu.2025.107560

Kremers, I. P., Van Giezen, A. E., Van Der Does, A. J. W., Van Dyck, R., & Spinhoven, Ph. (2007). Memory of childhood trauma before and after long-term psychological treatment of borderline personality disorder. *Journal of Behavior Therapy and Experimental Psychiatry*, *38*(1), 1–10. https://doi.org/10.1016/j.jbtep.2005.12.003

McKinney, C. M., Harris, T. R., & Caetano, R. (2009). Reliability of Self-Reported Childhood Physical Abuse by Adults and Factors Predictive of Inconsistent Reporting. *Violence and Victims*, *24*(5), 653–668. https://doi.org/10.1891/0886-6708.24.5.653

Mersky, J. P., Janczewski, C. E., & Topitzes, J. (2017). Rethinking the Measurement of Adversity: Moving Toward Second-Generation Research on Adverse Childhood Experiences. *Child Maltreatment*, *22*(1), 58–68. https://doi.org/10.1177/1077559516679513

Mesquita, C. S., & Maia, Â. C. (2018). What is told when the story is retold? Consistency of victimization reports in psychiatric patients. *Scandinavian Journal of Psychology*, *59*(3), 311–318. https://doi.org/10.1111/sjop.12437

Naicker, S. N., Norris, S. A., Mabaso, M., & Richter, L. M. (2017). An analysis of retrospective and repeat prospective reports of adverse childhood experiences from the South African Birth to Twenty Plus cohort. *PLOS ONE*, *12*(7), e0181522. https://doi.org/10.1371/journal.pone.0181522

Nelson, E. C., Lynskey, M. T., Heath, A. C., Madden, P. A. F., & Martin, N. G. (2010). A Family Study of Adult Twins with and without a History of Childhood Abuse: Stability of Retrospective Reports of Maltreatment and Associated Family Measures. *Twin Research and Human Genetics*, *13*(2), 121–130. https://doi.org/10.1375/twin.13.2.121

Okeke, N. L., Wilkinson, A. V., & Roberts, R. E. (2017). The Stability of Retrospective Child Sexual Abuse Reports and its Association With Problem Avoidance. *Journal of Child Sexual Abuse*, *26*(6), 677–691. https://doi.org/10.1080/10538712.2017.1307892

Paivio, S. C. (2001). Stability of retrospective self-reports of child abuse and neglect before and after therapy for child abuse issues☆. *Child Abuse & Neglect*, *25*(8), 1053–1068. https://doi.org/10.1016/S0145-2134(01)00256-3

Paivio, S. C., & Cramer, K. M. (2004). Factor structure and reliability of the Childhood Trauma Questionnaire in a Canadian undergraduate student sample. *Child Abuse & Neglect*, *28*(8), 889–904. https://doi.org/10.1016/j.chiabu.2004.01.011

Park, S. (2018). Reliability and Validity of the Early Trauma Inventory Self Report-Short Form among Korean Adolescents. *Journal of the Korean Academy of Child and Adolescent Psychiatry*, *29*(1), 2–6. https://doi.org/10.5765/jkacap.2018.29.1.2

Peng, C., Cheng, J., Rong, F., Wang, Y., & Yu, Y. (2023). Psychometric properties and normative data of the childhood trauma questionnaire-short form in Chinese adolescents. *Frontiers in Psychology*, *14*, 1130683. https://doi.org/10.3389/fpsyg.2023.1130683

Pereira Da Silva, S. S., & Da Costa Maia, Â. (2013). The Stability of Self-Reported Adverse Experiences in Childhood: A Longitudinal Study on Obesity. *Journal of*

Interpersonal Violence, *28*(10), 1989–2004. https://doi.org/10.1177/0886260512471077

Pinto, R., Correia, L., & Maia, Â. (2014). Assessing the Reliability of Retrospective Reports of Adverse Childhood Experiences among Adolescents with Documented Childhood Maltreatment. *Journal of Family Violence*, *29*(4), 431–438. https://doi.org/10.1007/s10896-014-9602-9

Plaza, A., Torres, A., Martin-Santos, R., Gelabert, E., Imaz, M. L., Navarro, P., Bremner, J. D., Valdes, M., & Garcia-Esteve, L. (2011). Validation and Test-Retest Reliability of Early Trauma Inventory in Spanish Postpartum Women. *Journal of Nervous & Mental Disease*, *199*(4), 280–285. https://doi.org/10.1097/NMD.0b013e31821245b9

Racine, N., Plamondon, A., Mcdonald, S., Tough, S., & Madigan, S. (2020). The Consistency of Maternal Childhood Abuse Reporting in Pregnancy and the Postpartum Period. *Journal of Women's Health*, *29*(4), 561–569. https://doi.org/10.1089/jwh.2019.7795

Shannon, C., Hanna, D., Tumelty, L., Waldron, D., Maguire, C., Mowlds, W., Meenagh, C., & Mulholland, C. (2016). Reliability of reports of childhood trauma in bipolar disorder: A test–retest study over 18 months. *Journal of Trauma & Dissociation*, *17*(4), 511–519. https://doi.org/10.1080/15299732.2016.1141147

Simpson, S., Phillips, L., Baksheev, G., Garner, B., Markulev, C., Phassouliotis, C., Alvarez-Jimenez, M., McGorry, P., & Bendall, S. (2019). Stability of retrospective self-reports of childhood trauma in first-episode psychosis. *Early Intervention in Psychiatry*, *13*(4), 908–913. https://doi.org/10.1111/eip.12700

Smith, J. P., & Thomas, D. (2003). Remembrances of Things Past: Test–Retest Reliability of Retrospective Migration Histories. *Journal of the Royal Statistical Society Series A: Statistics in Society*, *166*(1), 23–49. https://doi.org/10.1111/1467-985X.00257

Spinhoven, P., Bamelis, L., Haringsma, R., Molendijk, M., & Arntz, A. (2012). Consistency of reporting sexual and physical abuse during psychological treatment of personality disorder: An explorative study. *Journal of Behavior Therapy and Experimental Psychiatry*, *43*, S43–S50. https://doi.org/10.1016/j.jbtep.2011.02.005

Teicher, M. H., & Parigger, A. (2015). The 'Maltreatment and Abuse Chronology of Exposure' (MACE) Scale for the Retrospective Assessment of Abuse and Neglect During Development. *PLOS ONE*, *10*(2), e0117423. https://doi.org/10.1371/journal.pone.0117423

Wielaard, I., Stek, M. L., Comijs, H. C., & Rhebergen, D. (2018). Reliability of retrospective reports on childhood abuse and its determinants in older adults during a 6-year follow-up. *Journal of Psychiatric Research*, *105*, 9–16. https://doi.org/10.1016/j.jpsychires.2018.08.009

Xiang, Z., Liu, Z., Cao, H., Wu, Z., & Long, Y. (2021). Evaluation on Long-Term Test–Retest Reliability of the Short-Form Childhood Trauma Questionnaire in Patients with Schizophrenia. *Psychology Research and Behavior Management*, *Volume 14*, 1033–1040. https://doi.org/10.2147/PRBM.S316398

Yancura, L. A., & Aldwin, C. M. (2009). Stability and change in retrospective reports of childhood experiences over a 5-year period: Findings from the Davis Longitudinal Study. *Psychology and Aging*, *24*(3), 715–721. https://doi.org/10.1037/a0016203

Zanotti, D. C., Kaier, E., Vanasse, R., Davis, J. L., Strunk, K. C., & Cromer, L. D. (2018). An examination of the test–retest reliability of the ACE-SQ in a sample of college athletes. *Psychological Trauma: Theory, Research, Practice, and Policy*, *10*(5), 559–562. https://doi.org/10.1037/tra0000299

Zhang, J., Wu, Z., Chen, M., Gao, Y., Liu, Z., Long, Y., & Chen, X. (2024). Factor analysis and evaluation of one-year test-retest reliability of the 33-item Childhood Trauma Questionnaire in Chinese adolescents. *Frontiers in Psychology*, *15*, 1384807. https://doi.org/10.3389/fpsyg.2024.1384807
