# Supplementary material for: Stability of childhood maltreatment self-reports: a systematic review and meta-analysis
Source: Nat Ment Health. 2026 Jul 3;4(8):1315–28. doi: 10.1038/s44220-026-00677-7 (PMC13441976; doi:10.1038/s44220-026-00677-7)
Supplement: Supplementary file 2 [file 44220_2026_677_MOESM2_ESM.pdf]

nature portfolio

Corresponding author(s): Professor Andrea Danese

Last updated by author(s): Dec 18, 2025

Reporting Summary

Nature Portfolio wishes to improve the reproducibility of the work that we publish. This form provides structure for consistency and transparency in reporting. For further information on Nature Portfolio policies, see our Editorial Policies and the Editorial Policy Checklist.

Statistics

For all statistical analyses, confirm that the following items are present in the figure legend, table legend, main text, or Methods section.

n/a	Confirmed	
☐	☒	The exact sample size (n) for each experimental group/condition, given as a discrete number and unit of measurement
☐	☒	A statement on whether measurements were taken from distinct samples or whether the same sample was measured repeatedly
☐	☒	The statistical test(s) used AND whether they are one- or two-sided<br>*Only common tests should be described solely by name; describe more complex techniques in the Methods section.*
☐	☒	A description of all covariates tested
☒	☐	A description of any assumptions or corrections, such as tests of normality and adjustment for multiple comparisons
☐	☒	A full description of the statistical parameters including central tendency (e.g. means) or other basic estimates (e.g. regression coefficient) AND variation (e.g. standard deviation) or associated estimates of uncertainty (e.g. confidence intervals)
☐	☒	For null hypothesis testing, the test statistic (e.g. F, t, r) with confidence intervals, effect sizes, degrees of freedom and P value noted<br>*Give P values as exact values whenever suitable.*
☒	☐	For Bayesian analysis, information on the choice of priors and Markov chain Monte Carlo settings
☒	☐	For hierarchical and complex designs, identification of the appropriate level for tests and full reporting of outcomes
☐	☒	Estimates of effect sizes (e.g. Cohen's d, Pearson's r), indicating how they were calculated

Our web collection on statistics for biologists contains articles on many of the points above.

Software and code

Policy information about availability of computer code

Data collection	Studies were screened and selected for inclusion using Rayyan (https://www.rayyan.ai/)
Data analysis	Analyses were conducted using the Metafor package in RStudio (Version 2025.09.2+418).

For manuscripts utilizing custom algorithms or software that are central to the research but not yet described in published literature, software must be made available to editors and reviewers. We strongly encourage code deposition in a community repository (e.g. GitHub). See the Nature Portfolio guidelines for submitting code & software for further information.

Data

Policy information about availability of data

All manuscripts must include a data availability statement. This statement should provide the following information, where applicable:

- Accession codes, unique identifiers, or web links for publicly available datasets
- A description of any restrictions on data availability
- For clinical datasets or third party data, please ensure that the statement adheres to our policy

The script and data extraction table for the analysis are available at: https://github.com/oonaghcoleman/maltreatment-memory-stability.

Research involving human participants, their data, or biological material

Policy information about studies with human participants or human data. See also policy information about sex, gender (identity/presentation), and sexual orientation and race, ethnicity and racism.

Reporting on sex and gender	We report the sex distribution of each included study as the percentage of female participants, based on the information provided in the original publications (Table 1). We conducted moderation analysis examining whether stability estimates differed by sex composition of the sample.
Reporting on race, ethnicity, or other socially relevant groupings	Race and ethnicity were not examined in this meta-analysis because this information was not consistently reported across the included studies.
Population characteristics	We extracted and coded key population characteristics relevant to the analyses, including sex, age, study location, and sample type. Specifically, we recorded the sex distribution of each study (percentage of female participants) as well as the mean age of participants at baseline and follow-up. We documented the geographic location of each study by country and classified sample type as clinical, convenience, or population-representative. For clinical samples, we additionally recorded the primary type of psychopathology represented.
Recruitment	This study is a meta-analytic review and therefore did not involve direct recruitment of participants. Differences in sample type (clinical, convenience, or population-representative samples) were examined through moderation analyses to assess their potential impact on the meta-analytic estimate.
Ethics oversight	This study is a meta-analytic review and therefore ethics oversight does not apply.

Note that full information on the approval of the study protocol must also be provided in the manuscript.

Field-specific reporting

Please select the one below that is the best fit for your research. If you are not sure, read the appropriate sections before making your selection.

☐ Life sciences ☒ Behavioural & social sciences ☐ Ecological, evolutionary & environmental sciences

For a reference copy of the document with all sections, see nature.com/documents/nr-reporting-summary-flat.pdf

Behavioural & social sciences study design

All studies must disclose on these points even when the disclosure is negative.

Study description	Systematic review and meta-analysis
Research sample	The systematic search identified 49 studies examining the stability of self-reports of child maltreatment (Table 1; Supplementary Figure 2). The studies were based on 49 cohorts (although one study used two different cohorts, and one cohort was used by two studies). There were 92,807 individuals assessed at baseline, and 38,332 of these individuals were retained at follow-up, with an average attrition rate of 59%. Participants were 60.4% female, the average age at baseline assessment was 32.2 years (range=55.3, median=33.0), the average age at follow-up assessment was 34.4 years, and the average follow-up interval was 2.4 years (123.6 weeks).
Sampling strategy	Inclusion criteria consisted of peer-reviewed longitudinal studies that measured the stability of self-reports of child maltreatment and were written in English. Using Rayyan, titles and abstracts of all articles captured by the search were independently screened by one doctoral student (OC) and independent raters with undergraduate degree training who were blind to each other's decisions. Full texts of potentially eligible studies were then screened independently following the same procedure. Inconsistencies were discussed and resolved with the senior author (AD). The screening process yielded a final sample of 49 studies for inclusion in the meta-analysis.
Data collection	Data were extracted independently by one doctoral student (OC) and double-entered by an independent rater. Inconsistencies were discussed and resolved in consensus meetings. Data extraction included stability estimate(s), exposure type(s), sample characteristics (population representative, convenience, or clinical sampling), measure type (interview vs. questionnaire), demographic factors (sex, age at baseline and follow-up assessment), time interval between assessments, attrition rate, study quality characteristics (population representativeness of the sample, attrition rate, whether the measures were validated, and whether the study controlled for the effects of sociodemographic or clinical variables on the stability estimate). For clinical samples, we also extracted data on the type of psychopathology and the presence of an intervention between baseline and follow-up
Timing	We screened and included studies from database inception to 01/10/25.
Data exclusions	Exclusion criteria included studies published in a language other than English, studies that were not peer-reviewed, studies in which maltreatment was assessed at only one time point, studies that did not use self-report measures of maltreatment, studies that did

	not use the same assessment type across time points, studies that measured changes in exposure rather than changes in reporting, and studies with a time interval of less than two months between assessments.
Non-participation	There were 92,807 individuals assessed at baseline, and 38,332 of these individuals were retained at follow-up, with an average attrition rate of 59%. Attrition did not moderate the meta-analytic estimate in moderation analysis.
Randomization	This study is a meta-analytic review of observational studies and therefore did not involve allocation of participants to experimental groups.

Reporting for specific materials, systems and methods

We require information from authors about some types of materials, experimental systems and methods used in many studies. Here, indicate whether each material, system or method listed is relevant to your study. If you are not sure if a list item applies to your research, read the appropriate section before selecting a response.

Materials & experimental systems

n/a	Involved in the study
☒	☐ Antibodies
☒	☐ Eukaryotic cell lines
☒	☐ Palaeontology and archaeology
☒	☐ Animals and other organisms
☒	☐ Clinical data
☒	☐ Dual use research of concern
☒	☐ Plants

Methods

n/a	Involved in the study
☒	☐ ChIP-seq
☒	☐ Flow cytometry
☒	☐ MRI-based neuroimaging

Plants

Seed stocks	N/A
Novel plant genotypes	N/A
Authentication	N/A
