# Supplementary material for: Stability of childhood maltreatment self-reports: a systematic review and meta-analysis
Source: Nat Ment Health. 2026 Jul 3;4(8):1315–28. doi: 10.1038/s44220-026-00677-7 (PMC13441976; doi:10.1038/s44220-026-00677-7)
Supplement: Supplementary file 3 [file 44220_2026_677_MOESM3_ESM.pdf]

nature portfolio

Peer Review File

Stability of childhood maltreatment self-reports: A systematic review and meta-analysis

Corresponding Author: Professor Andrea Danese

This file contains all reviewer reports in order by version, followed by all author rebuttals in order by version.

Version 1:

Reviewer comments:

Reviewer #1

(Remarks to the Author)
General Comment

This manuscript addresses an important and timely topic: the stability of retrospective self-reports of childhood maltreatment. Given the widespread reliance on such measures in both research and clinical practice, evaluating their reliability has clear practical and theoretical significance. The authors present a well-conducted systematic review and meta-analysis, employing a sound methodological framework that includes appropriate database coverage, thoughtful harmonization of effect sizes, and standard meta-analytic techniques. The findings are both compelling and relevant, and the conclusions are appropriately grounded in the evidence presented. Overall, the study makes a meaningful contribution to the literature and will be of interest to researchers and practitioners concerned with the assessment of childhood adversity. That said, I have two major comments: one concerns the search strategies, and the other concerns the methodology used in the meta-analysis.

Major Comment #1: Search Strategies

Database coverage

The manuscript relies on three databases: Medline, PsycInfo, and Embase. Together these databases provide a comprehensive coverage of medical, psychological, and psychiatric literature. However, the search strategy may benefit from a broader interdisciplinary reach.

Specifically, the current database selection may underrepresent studies from adjacent fields such as nursing, social work, education, and criminology, where relevant research on childhood maltreatment and trauma assessment is also published. For example, CINAHL could capture nursing and allied health literature, while Scopus or Web of Science may identify interdisciplinary studies and citation-linked research not indexed in the core databases. Additionally, grey literature sources (e.g., ProQuest Dissertations & Theses) may contain unpublished longitudinal studies or conference proceedings relevant to the topic.

Search terms for maltreatment

The current search strategy includes core maltreatment-related terms such as abuse, neglect, trauma, and adverse childhood experiences, which are central to psychology and psychiatry. However, it omits broader descriptors such as violence, victimization, interpersonal violence, and exposure to domestic violence, which are frequently used in adjacent disciplines. This may not capture relevant studies outside the mainstream psychology literature, thus limit the comprehensiveness of the review.

Search terms for reliability and stability

The current search strategy for reliability and stability includes key terms such as test–retest, reproducibility, and ICC, but omits other commonly used descriptors like agreement, validity, measurement error, and concordance.

Recommendation:

To improve the comprehensiveness of the review, I suggest that the authors consider expanding both the database coverage and search terms. Importantly, the authors might assess whether these additions lead to substantive changes in the set of included studies. If re-running the search is feasible, a brief analysis of the impact of expanded sources and terms on study inclusion would be highly informative. If not feasible, the manuscript should explicitly acknowledge these limitations and discuss their potential implications for the scope and representativeness of the findings.

Major Comment #2: Effect Size Harmonization and Use of Tetrachoric Correlation

The authors employs a rigorous and transparent analytic pipeline for harmonizing effect sizes across studies. The authors convert diverse stability metrics—including kappa, ICC, and test–retest correlations—into tetrachoric correlations, apply Fisher's z-transformation for variance stabilization, calculate meta-analytic weights, and back-transform results for interpretation. This approach is methodologically sound and well-documented.

However, several conceptual and statistical assumptions underlying this strategy warrant closer scrutiny:

Metric equivalence: Harmonizing kappa, ICC, and Pearson correlations assumes these metrics are interchangeable reflections of a common latent construct. This may oversimplify important differences in their conceptual foundations and statistical behavior.

Calibration from kappa to tetrachoric: Kappa and tetrachoric correlation are not directly equivalent. Kappa adjusts for chance agreement in categorical data, while tetrachoric assumes threshold latent traits. The calibration model used to convert kappa values may introduce bias, especially in studies with low prevalence or skewed distributions.

Latent distribution assumptions: Tetrachoric correlation assumes binary outcomes reflect cutoffs on normally distributed latent variables. This assumption may be reasonable for some types of maltreatment (e.g., physical abuse or neglect), but not others (e.g., sexual abuse). In particular, since sexual abuse is often experienced as a discrete event rather than a gradation of intensity, the construct may not map onto a continuous latent continuum.

Sparse data and small samples: Tetrachoric estimates can be unstable when derived from small or unbalanced 2×2 tables, potentially inflating variance or biasing effect sizes.

Recommendation: To address these concerns, I recommend that the authors conduct sensitivity analyses comparing results across alternative effect size metrics—such as raw kappas, phi coefficients, or excluding converted kappas. Reporting whether the main conclusions hold across these specifications would enhance confidence in the robustness of the findings. Additionally, a brief discussion of the conceptual limitations of tetrachoric correlation in the context of self-reported maltreatment would improve transparency and interpretability.

In sum, this manuscript presents a rigorous and timely meta-analysis with clear relevance to both research and clinical practice. While several methodological aspects—particularly the search strategies and effect size harmonization—would benefit from clarification or additional sensitivity analyses, these concerns are addressable and do not undermine the overall contribution of the work. I recommend minor revision, as these improvements will enhance the transparency and robustness of the work.

(Remarks on code availability)
I have not run the code or tried to reproduce the analyses conducted, but the overall analysis strategy appears to be sound (but see my second major comment).

Reviewer #2

(Remarks to the Author)
Summary of key results
The meta-analysis examined the stability of childhood maltreatment measures and found generally large stability, with age (among others) emerging as a key and significant moderator. The rationale for including moderators is clearly explained, and the methods are robust and comprehensive. Key limitations of the reviewed studies and the approach taken have been acknowledge, eg attrition, confound of age with recall period, etc.

Originality and significance

The study is original and significant in its synthesis of the literature and in highlighting important moderators, such as age, that can inform practice. Its findings have relevance beyond clinical settings, including social care and legal contexts where questions of stability and accuracy of maltreatment reports (such as concerns about false memories or accounts) are often debated. Expanding on these broader implications would strengthen the manuscript and ensure it is not perceived as primarily a psychometric or epidemiological exercise.

Data and methodology
The study demonstrates careful methodology, but certain aspects require clarification. For example, while quality of studies

was considered as a moderator, it is not clear which assessment tool was used or whether it was the most appropriate. The choice to prioritize shorter time intervals in analysis may also bias results toward apparent stability, whereas longer intervals may be more informative in understanding clinical and developmental implications.

Appropriate use of statistics and treatment of uncertainties
The statistical approach appears sound, but some caution is warranted in interpretation. For example, trends observed for female participants were not significant – is this a power issue or absence of an effect? The consideration of age as a moderator is interesting, particularly in the context of adolescent malleability, but the analysis could further explore alternative definitions of adolescence extending into the early twenties, which are increasingly relevant for research and early intervention.

Conclusions
The conclusions are generally supported by the data, but some interpretations risk overstating clinical implications. While the stability of maltreatment measures is confirmed, presenting the results as directly informing clinical practice may oversimplify the issue. There is an overreliance on reappraisal as a mechanistic explanation for stability, which is then used to justify therapeutic approaches (e.g., trauma-focused CBT). Other evidence-based interventions, such as EMDR or imagery rescripting, are also relevant, and the study does not distinguish between mechanisms, so it is premature to make specific intervention recommendations. In fact, more research is needed to clarify the cognitive mechanisms underlying the stability of self-reported maltreatment – this isn't highlighted; suggestions for future avenues would be useful. There are also potential risks of overfocusing on maltreatment experiences, particularly in youth, where recall and appraisals are highly malleable. It is unclear whether the authors are implying that all young people with a maltreatment history should receive reappraisal-focused interventions, even when the primary clinical presentation is unrelated, such as panic disorder or OCD.

Suggested improvements
Future revisions could clarify the choice of quality assessment tools and provide justification for time intervals used in analysis. The literature search should be updated as it is almost 2 years since the search. Considering alternative definitions of adolescence and reflecting on the implications of malleability for both research and practice beyond the clinic (eg social care, legal contexts) would strengthen the manuscript, as the issue of 'stability' is perhaps more relevant there. A more balanced and open perspective on underlying mechanisms (and therefore the treatment implications) would be recommended.

References
Fine

Clarity and context
The manuscript is clearly written, with an informative abstract and introduction. The rationale for the study is well justified, and the methodology is presented in detail. However, the discussion could better contextualise findings within a wider range of applied contexts where the measurement stability issue is perhaps more salient.

(Remarks on code availability)

Reviewer #3

(Remarks to the Author)

This manuscript presents a comprehensive meta-analysis on the temporal stability of retrospective self-reports of childhood maltreatment across 46 studies. The topic is relevant, the data source appropriate and the manuscript is well-written and provides valuable insights. While the topic is not entirely novel, the scope and synthesis are original and significant.
While I overall commend the authors to their approach, I think the manuscript needs revision in a number of aspects to improve precision, transparency, and appropriateness of interpretation. Please note that I am not an expert in the methodology of meta-analysis, such that I advise the editor to additionally invite a reviewer with explicit expertise in this field.

Abstract
• Line 42: The claim that "these findings challenge the prevailing view that retrospective self-reports are inherently unstable" should be substantiated. It is unclear whether this view is truly prevailing.
• Line 44: The statement that "reshaping trauma-related appraisals may require deliberate intervention and might be most effective during childhood" appears like a wide stretch. Please clarify how this conclusion is derived from the presented results.

Introduction
• Line 71: Please clarify what is meant by maltreatment being a "modifiable" risk factor.
• Line 104: The mechanisms proposed to explain higher stability in clinical samples (e.g., mood congruency, rumination, causal search bias) are mentioned but not sufficiently explained. Consider integrating the more detailed rationale from the discussion section here.

Methods
• Please explain why interrater agreement was measured using percentage overlap instead of using common metrics for

interrater agreement.
• There is no need to mention author status or prior experience (e.g., “doctoral student”).
• From line 168 onwards, variables are listed as “e.g.”. Please provide a complete list of all extracted variables and all analyses conducted.
• The analysis of psychopathology type as a moderator appears in the discussion but is not described in the methods. Please include it and clarify whether it was preregistered.
• The exclusion of two studies due to potential new experiences in under-18 participants should be considered for all analyses (instead of just the moderator analyses). Alternatively, you could test whether the analyses yield different results without these two studies and report as robustness checks in the supplement.
• The dichotomization of age at 18 years seems problematic to me. This cutoff lacks theoretical justification (e.g., regarding cognitive processes) and reduces information. If retained, mark it clearly as exploratory and not preregistered. Also, please clarify the age distribution within the 0–18 group (e.g., proportion of children vs. adolescents).

Results
• Descriptive statistics for key variables (e.g., maltreatment types, study quality) should be included in the main text, not only in the supplement.
• Age should be described more thoroughly: include range, median, and proportion of participants under 18 and/or show the distribution visually.
• Line 242: Please explain the large number of effect sizes (224) relative to the number of studies (46). Clarify what these effect sizes represent.
• Justify the use of correlation coefficients (r) instead of standard test-retest reliability measures. It would be helpful if you also report the effect size metric used in the original studies.
• Please additionally include a more interpretable measure such as percentage overlap (between the two time points), especially since percentage overlap is already partially used in figures and the discussion (e.g., line 318), but lacking in the results section. Also report omission vs. commission rates in the results section to better understand the non-overlapping part of the reports.
• Ensure consistent reporting of statistical parameters across moderator analyses (e.g., neglect vs. abuse, representative vs. non-representative samples).
• Avoid reporting “trends for significance.” Please use a predefined significance threshold and stick to it.
• Clarify which analyses were preregistered and which were added post hoc (e.g., logarithmic vs. linear age terms).
• Line 283: Please provide inferential statistics for the comparison between younger and older participants.
• Line 291: Report the effect size, not just the p-value, for the interaction term.
• Table 2: Clarify the meaning of the correlation column for continuous moderators (e.g., age, sex, attrition).
• Table 3 is not referenced in the text. Please integrate and explain.

Discussion
• Line 318: The “20%” divergence in reporting comes out of the blue and should be introduced earlier in the results.
• Line 334: Consider whether lower certainty about experiences may contribute to lower stability in population-based samples.
• Line 350: you mention that „Causal search bias, where individuals reinterpret past experiences to explain their distress or difficulties, could also increase consistency by reinforcing negative interpretations of memories over time”. Could causal search bias also lead to inconsistency of reports if interpretations change over time?
• Line 355: The phrase “Of note” is ambiguous. Clarify whether these findings were preregistered.
• Line 360: The sentence “Because low stability adds to measurement error…” is unclear in its continuation. Please revise.
• Line 362: Clarify what neglect is being compared to in “lower agreement with prospective measures.”
• Line 363: Avoid using “on one hand… on the other hand…” when both arguments point in the same direction.
• Line 363: The claim that abuse is a stronger emotional experience than neglect should be supported by evidence.
• Line 373: Specify that “first recalled maltreatment in childhood” refers to the first measurement point of this study, not the first recall in general.
• Line 376: The analysis that excluded the two ambivalent studies likely provides more reliable results. Please reframe the interpretation accordingly.
• Line 385: „Children’s and adolescents’ memories may be more malleable and susceptible to reinterpretation ... In contrast, adults’ memories may be more stable over time because of repeated rehearsal and integration into a coherent life narrative“ - Consider discussing that on the other hand, maltreatment events are temporally more distant for adults, which may affect memory accuracy negatively.
• Line 390: The comparison to personality traits and cognitive abilities does not seem very informative or appropriate to me. These constructs (abilities and traits) differ fundamentally from event memory. (also see line 411). CSA experiences should not be treated as a personality trait.
• Line 398: “„The findings should also be interpreted within the context of research studies similar to those included in the meta-analyses and may not generalise to other contexts (e.g., to disclosures that reach the attention of authorities).” Please expand on generalizability. Specify which contexts the findings apply to and how these differ from cases reported to authorities.
• Line 404: „While no significant effect was detected in our moderation analysis, attrition could still introduce bias“ – Please mention the potential direction of bias here.
• Line 451: The therapeutic implications are not clearly supported by the data. Please clarify the connection and avoid overinterpretation. Also: Why should interventions be more usefull in childhood, if reports have shown to be more inconsistent in childhood?

General Comments

• Clarify how original studies handled ambivalent or uncertain reports, e.g., participants reporting being unsure about having experienced maltreatment. How were they forced into a dichothomeous variable? Was certainty about responses measured?
• Did you examine potential changes of maltreatment type between the two timepoints? (i.e., stable overall report but changing subtype)?
• The interpretation of r = .79 as "very stable" should be contextualized. (even a correlation of r = .62 is considered „high stability", line 397). I would appreciate a more differeniated interpretation. What level of stability is expected by yourself, by lay people or by experts? How does this compare to stability of other memory constructs?
• Discuss limitations of dichotomous classification (maltreatment vs. no maltreatment) in terms of memory and psychometrically / statistically.
• Table 3 shows no effect of interventions on memory stability. Could this challenge assumptions about repression and memory recovery in therapy? This would be worth mentioning.
• Reported maltreatment experiences are treated as „memories" in the manuscript. Consider referring to "reports" rather than "memories," and discuss the distinction between self-report and factual accuracy.
• Line 434: I disagree with your claim that retrospective measures of maltreatment can be treated as trait-like features. We still don't know if the reports are based on actual experiences, just because their are reported consistently (also considering former studies by your research team on inconsistent reporting).
• I find this conclusion hard to follow: "It also suggests that the lack of agreement between prospective and retrospective measures is unlikely to be driven by shifting retrospective reports over time, and instead may be driven by stable individual differences between those identified as maltreated by each measure". I dont see how this conclusion can be drawn from your data. Reports could be consistent, but false. Please clarify.
• Also, how does the interpretation of „stable individual differences", relates to the findings of your former studies on inconsistent reporting of maltreatment between childhood and adulthood? As an alternative explanation, there may be inconsistent reporting during childhood until people stick to their report, irrespective of truth-status.
• Your work would overall benefit if you discuss your findings more in light of other work by Danese and colleagues on inconsistencies between prospective and retrospective reports, and between childhood and adolescent reporting.
• I also think you could somewhat enrich the introduction section. Maybe my former comments provide inspiration for further arguments to include. But this is probably more a matter of taste.

Thank you for the opportunity to review this manuscript.
Elsa Gewehr

(Remarks on code availability)
Please note that I am not an expert in the methodology of meta-analysis, such that I advise the editor to additionally invite a reviewer with explicit expertise in this field.

Reviewer #4

(Remarks to the Author)
Review: Nature Mental Health
Manuscript Number: NATMH-25-0898A

In the manuscript the authors report a systematic review on a highly relevant and interesting area. The meta-analysis includes 46 studies (37,880 participants) investigating the temporal stability of retrospective self-reports of childhood maltreatment. The authors report an overall high stability over an average 2.5-year interval, while noting considerable heterogeneity across studies. Stability varied by maltreatment type (neglect < abuse), sample type (population-based < clinical), and age (lower in younger participants, particularly under 18 years). In my opinion, this study addresses an important gap in the literature and provides knowledge with clear implications for both research methodology and clinical practice. The study comes with several strengths, which I will list below. However, I also have some concerns and suggestions which I believe, if taken into account, will improve the manuscript.

Strengths:

- Novelty and significance: To my knowledge, this is the first quantitative synthesis of retrospective
- The study comes with rigorous methodology.
- Comprehensive analyses: The use of multilevel models, sensitivity test, and moderation analyses is impressive and allows nuanced interpretation.
- Clarity of reporting: The manuscript is well structured, with transparent methods and clear figures.

Major concerns/suggestions:

• Conceptual framing: The introduction could and should more explicitly express how findings inform the validity of retrospective self-reports as measures of exposure vs. memory accuracy. With this I particularly mean: Are we interpreting stability as evidence for reliability of reports, or as insights into memory processes? Clarifying this conceptual distinction would strengthen the discussion of the results.

• Interpretation of heterogeneity: The I^2 value is extremely high (>98%). While moderation analyses identify some meaningful moderators, residual heterogeneity remains substantial. It would be useful for the authors to discuss whether this reflects measurement variability, true differences in recall processes, or both.

• Age/developmental issues:
1) The age x interval interaction is one of the most compelling findings, yet the main text presents it rather briefly. The introduction should address developmental theories on autobiographical memory (e.g., narrative identity, recall of traumatic periods/events, consolidation) to a greater extent. (See e.g., Bauer et al., Hjuler et al., Fivush et al.,). This is essential to ground the study results more strongly in cognitive developmental science.
2) When referring to age >18, the authors write 'children', however, the WHO definition of children over 10 years of age is 'adolescents'. I recommend that you consider which term to use, and at least to explain what is meant by 'children' in your study.
3) A major concern is also that the authors cut-off when referring to children is 18 years of age. Considering the massive development on autobiographical development during childhood (with regards to encoding, consolidation and recall), it would be valuable to consider a lower-age cut-off. For instance 12-15-years of age; 15-18-years of age and above 18 years of age.
4) In line with the above; the authors discuss 're-shaping trauma related memories in childhood'. However, with a cut-off of 18-years of age, such a discussion should be considered carefully.

• Time interval and measure types:
1) The study includes studies with a time interval of two months or longer. What is the rationale for this time interval? This must be explained in the manuscript.
2) In addition, the authors write that 'for studies that measured stability at multiple time points, the longest interval was selected by default', Please explain the rational behind this choice of selection as well.
3) While interviews vs questionnaires did not moderate stability, could measure length/detail (e.g., single-item vs. multi-item scales) be a relevant factor? If such data are unavailable, this limitation should be acknowledged.

• Neglect vs. abuse: The finding of lower stability for neglect deserves further unpacking. The authors suggest emotional salience as an explanation, but neglect also differs conceptually (absence of care rather than presence of harmful acts). This distinction could be elaborated with reference to how neglects is operationalized and experienced. Also, when explaining the different types of child maltreatment, examples could be helpful. Please make sure to provide sufficient references.

• Clinical implications: The discussion highlights risks of misclassification, but clinical applications (e.g., use in assessment, legal testimony, trauma-informed care) could be more directly addressed. How should practitioners interpret a high overall stability yet 20% change in reports?

Minor Comments

1. Some references are duplicated in the text (e.g., Coleman et al., 2024 cited twice in the same line). A careful proofread is needed.
2. Figures and supplementary tables are well designed, but figure legends could briefly restate the key finding for accessibility.
3. In the methods, clarify whether quality ratings were incorporated into sensitivity analyses or only descriptively reported.
4. Reporting of participant demographics: if available, indicate diversity (ethnicity, socioeconomic background) across studies, as these may shape recall stability.
5. Wording: the phrase "non-negligible proportion (20%)" could be rephrased for clarity ("approximately one in five participants changed their report").
6. Please provide kappa values when reporting inter-rater agreement results.
7. Line 175: Please explain the rationale.
8. Line 395: "The fining should be interpreted….." I believe a 's' is missing in 'finding'?
9. Line 395: "provides reassurance" – I would recommend to write this more cautiously.
10. Line 442: When addressing that interventions might be more beneficial if delivered early in life, please keep in mind that psychiatric diagnosis because of maltreatment are typically diagnosed later in life.

Recommendation
Based on my above comments, I recommend major revision, primarily to expand the theoretical framing and deepen the discussion of heterogeneity, developmental processes, and clinical implications.

(Remarks on code availability)

Version 2:

Reviewer comments:

Reviewer #1

(Remarks to the Author)

The authors have provided thoughtful responses to the questions and recommendations raised in my initial review. The revisions adequately address the concerns I identified, and the manuscript has been improved accordingly. I am satisfied with the revised manuscript and recommend acceptance for publication."

(Remarks on code availability)

Reviewer #2

(Remarks to the Author)
I am satisfied with all their rebuttal to my points

(Remarks on code availability)
NA

Reviewer #3

(Remarks to the Author)

Dear Editor and authors,
I would like to express my appreciation for the thorough and constructive way in which the authors engaged with my and the other reviewers comments. They have made substantial efforts to revise the paper, leading to a manuscript with substantially improved precision and clarity.
The authors have addressed my suggestions appropriately or provided reasonable explanations when they took a different position.
I therefore recommend to accept and publish the manuscript in its current form.
Kind regards,
Elsa Gewehr

(Remarks on code availability)

Open Access This Peer Review File is licensed under a Creative Commons Attribution 4.0 International License, which permits use, sharing, adaptation, distribution and reproduction in any medium or format, as long as you give appropriate credit to the original author(s) and the source, provide a link to the Creative Commons license, and indicate if changes were made.
In cases where reviewers are anonymous, credit should be given to 'Anonymous Referee' and the source.
The images or other third party material in this Peer Review File are included in the article’s Creative Commons license, unless indicated otherwise in a credit line to the material. If material is not included in the article’s Creative Commons license and your intended use is not permitted by statutory regulation or exceeds the permitted use, you will need to obtain permission directly from the copyright holder.
To view a copy of this license, visit https://creativecommons.org/licenses/by/4.0/

Reviewer #1:

Question #1.1: General Comment

This manuscript addresses an important and timely topic: the stability of retrospective self-reports of childhood maltreatment. Given the widespread reliance on such measures in both research and clinical practice, evaluating their reliability has clear practical and theoretical significance. The authors present a well-conducted systematic review and meta-analysis, employing a sound methodological framework that includes appropriate database coverage, thoughtful harmonization of effect sizes, and standard meta-analytic techniques. The findings are both compelling and relevant, and the conclusions are appropriately grounded in the evidence presented. Overall, the study makes a meaningful contribution to the literature and will be of interest to researchers and practitioners concerned with the assessment of childhood adversity. That said, I have two major comments: one concerns the search strategies, and the other concerns the methodology used in the meta-analysis.

Reply #1.1: We thank Reviewer 1 for their recognition of the importance of the topic, the positive evaluation of the manuscript, and the thoughtful suggestions below.

Q#1.2: Major Comment #1: Search Strategies

Database coverage

The manuscript relies on three databases: Medline, PsycInfo, and Embase. Together these databases provide a comprehensive coverage of medical, psychological, and psychiatric literature. However, the search strategy may benefit from a broader interdisciplinary reach.

Specifically, the current database selection may underrepresent studies from adjacent fields such as nursing, social work, education, and criminology, where relevant research on childhood maltreatment and trauma assessment is also published. For example, CINAHL could capture nursing and allied health literature, while Scopus or Web of Science may identify interdisciplinary studies and citation-linked research not indexed in the core databases. Additionally, grey literature sources (e.g., ProQuest Dissertations & Theses) may contain unpublished longitudinal studies or conference proceedings relevant to the topic.

Search terms for maltreatment

The current search strategy includes core maltreatment-related terms such as abuse, neglect, trauma, and adverse childhood experiences, which are central to psychology and psychiatry. However, it omits broader descriptors such as violence, victimization, interpersonal violence, and exposure to domestic violence, which are frequently used in adjacent disciplines. This may not capture relevant studies outside the mainstream psychology literature, thus limit the comprehensiveness of the review.

Search terms for reliability and stability

The current search strategy for reliability and stability includes key terms such as test–retest, reproducibility, and ICC, but omits other commonly used descriptors like agreement, validity, measurement error, and concordance.

Recommendation:

To improve the comprehensiveness of the review, I suggest that the authors consider expanding both the database coverage and search terms. Importantly, the authors might assess whether these additions lead to substantive changes in the set of included studies. If re-running the search is feasible, a brief analysis of the impact of expanded sources and terms on study inclusion would be highly informative. If not feasible, the manuscript should explicitly acknowledge these limitations and discuss their potential implications for the scope and representativeness of the findings.

R#1.2: We appreciate Reviewer 1's thoughtful and comprehensive feedback on the database selection and search terms. We agree that an expanded search strategy could further expand the breadth of studies identified. However, these additional databases and terms were not included in our pre-registered protocol, and further searches would require significant time investment with only tentative benefits. To avoid post-hoc changes to our methods and delays, we opted not to expand the scope of our searches. To address Reviewer 1's suggestions, we have now added a discussion of this limitation on page 12 of the revised manuscript draft, outlining the implications for the scope and representativeness of the findings. Instead, to update our searches, we have re-run the searches to include studies published between 1 January 2024 and the present, identifying three additional studies that have been incorporated into the meta-analysis. The overall stability estimate remained unchanged, and most moderation results were consistent, although follow-up age became a statistically significant moderator of stability. We hope the updated searches strengthen confidence in the robustness

and current relevance of the findings, and that the added limitation provides a more balanced interpretation of the findings.

Q#1.3: Major Comment #2: Effect Size Harmonization and Use of Tetrachoric Correlation

The authors employ a rigorous and transparent analytic pipeline for harmonizing effect sizes across studies. The authors convert diverse stability metrics—including kappa, ICC, and test–retest correlations—into tetrachoric correlations, apply Fisher's z-transformation for variance stabilization, calculate meta-analytic weights, and back-transform results for interpretation. This approach is methodologically sound and well-documented.

However, several conceptual and statistical assumptions underlying this strategy warrant closer scrutiny:

Metric equivalence: Harmonizing kappa, ICC, and Pearson correlations assumes these metrics are interchangeable reflections of a common latent construct. This may oversimplify important differences in their conceptual foundations and statistical behavior.

Calibration from kappa to tetrachoric: Kappa and tetrachoric correlation are not directly equivalent. Kappa adjusts for chance agreement in categorical data, while tetrachoric assumes threshold latent traits. The calibration model used to convert kappa values may introduce bias, especially in studies with low prevalence or skewed distributions.

Latent distribution assumptions: Tetrachoric correlation assumes binary outcomes reflect cutoffs on normally distributed latent variables. This assumption may be reasonable for some types of maltreatment (e.g., physical abuse or neglect), but not others (e.g., sexual abuse). In particular, since sexual abuse is often experienced as a discrete event rather than a gradation of intensity, the construct may not map onto a continuous latent continuum.

Sparse data and small samples: Tetrachoric estimates can be unstable when derived from small or unbalanced 2×2 tables, potentially inflating variance or biasing effect sizes.

Recommendation: To address these concerns, I recommend that the authors conduct sensitivity analyses comparing results across alternative effect size metrics—such as raw kappas, phi coefficients, or excluding converted kappas. Reporting whether the

main conclusions hold across these specifications would enhance confidence in the robustness of the findings. Additionally, a brief discussion of the conceptual limitations of tetrachoric correlation in the context of self-reported maltreatment would improve transparency and interpretability.

R#1.3: We are grateful for this thoughtful evaluation of the challenges involved in harmonizing and converting multiple effect size types. First, we must apologise, as we identified an inaccuracy in our description of the effect size conversion in the main text. On page 5, we had stated that different types of stability effect sizes were harmonized to tetrachoric correlations; however, this was incorrect. The correct description was provided in the Supplementary Materials. Studies reported a range of effect size types, including Pearson's correlations, Spearman's correlations, intraclass correlation coefficients (ICCs), and Cohen's kappa coefficients. We harmonised all stability estimates onto the correlational scale, converting kappa coefficients to tetrachoric correlations, while all other correlational effect sizes were retained as originally reported. We have now amended the main text on page 5 to accurately reflect this process.

We agree with Reviewer 1 that the calibration model used to convert kappa values to tetrachoric correlations may introduce some bias--and indeed had similar concerns. To address those concerns, in the original manuscript draft, we undertook a sensitivity analysis excluding the 34 effect sizes from 11 studies where tetrachoric correlations were estimated using this model. The meta-analytic findings remained very similar (r=0.79, CI=0.74, 0.83), suggesting that our results are robust to this issue. A description of this analysis can be found in the second paragraph on page 7 of the revised manuscript draft.

In response to Reviewer 1's suggestion, we additionally conducted a moderation analysis to examine whether stability estimates differed by effect size type. This analysis yielded a significant result (Q for moderation=14.1, p=0.001), with ICCs being significantly higher (k=17; r=0.86, CI=0.82,0.89) than both tetrachoric (k=24; r=0.75, CI=0.69, 0.81) and Pearson's/Spearman's correlations (k=10; r=0.72, CI=0.59, 0.81). Although the differences are statistically significant, the effect sizes are qualitatively similar (all large) and support the same conclusion of high stability. We have added a description of this to the Methods and Results section, and a discussion of the findings in the Limitations section.

Q#1.4: In sum, this manuscript presents a rigorous and timely meta-analysis with clear relevance to both research and clinical practice. While several methodological aspects—particularly the search strategies and effect size harmonization—would

benefit from clarification or additional sensitivity analyses, these concerns are addressable and do not undermine the overall contribution of the work. I recommend minor revision, as these improvements will enhance the transparency and robustness of the work.

R#1.4: We thank Reviewer 1 for the thoughtful suggestions above

<u>Reviewer #2:</u>

Q#2.1: Summary of key results
The meta-analysis examined the stability of childhood maltreatment measures and found generally large stability, with age (among others) emerging as a key and significant moderator. The rationale for including moderators is clearly explained, and the methods are robust and comprehensive. Key limitations of the reviewed studies and the approach taken have been acknowledge, eg attrition, confound of age with recall period, etc.

R#2.1: We thank Reviewer 2 for their positive evaluation of this paper and the helpful suggestions below.

Q#2.2: Originality and significance

The study is original and significant in its synthesis of the literature and in highlighting important moderators, such as age, that can inform practice. Its findings have relevance beyond clinical settings, including social care and legal contexts where questions of stability and accuracy of maltreatment reports (such as concerns about false memories or accounts) are often debated. Expanding on these broader implications would strengthen the manuscript and ensure it is not perceived as primarily a psychometric or epidemiological exercise.

R#2.2: We thank Reviewer 2 for raising this important point. We agree that the findings have relevance to debates about the stability and accuracy of maltreatment reports in legal and social care contexts, and we have expanded on this in the Discussion (pages 12-13). However, we also note that implications for practice should be considered with caution, given the well-documented gap between research settings and real-world reporting contexts. We hope that the extended discussion provides greater clarity on the originality and contribution of the paper's findings

Q#2.3: Data and methodology
The study demonstrates careful methodology, but certain aspects require clarification. For example, while quality of studies was considered as a moderator, it is not clear which assessment tool was used or whether it was the most appropriate. The choice to prioritize shorter time intervals in analysis may also bias results toward apparent stability, whereas longer intervals may be more informative in understanding clinical and developmental implications.

R#2.3: We thank Reviewer 2 for the opportunity to clarify this point. No standard tool exists for assessing study quality in research specifically focusing on test–retest reliability. To develop a quality assessment tool, adapted quality assessment items from a meta-analysis with a similar focus (Kobsar et al., 2020, Journal of NeuroEngineering and Rehabilitation).

We also wish to clarify that, as described on page 5, we prioritized longer rather than shorter follow-up intervals, as our focus was on memory-related effects that we expected to emerge and become more pronounced over longer time periods. We have now added an explicit rationale for this decision. The only exception was when a shorter interval provided a larger number of effect sizes for inclusion in the meta-analysis, in which case the shorter interval was selected, as noted on page 5.

Q#2.4: Appropriate use of statistics and treatment of uncertainties
The statistical approach appears sound, but some caution is warranted in interpretation. For example, trends observed for female participants were not significant – is this a power issue or absence of an effect? The consideration of age as a moderator is interesting, particularly in the context of adolescent malleability, but the analysis could further explore alternative definitions of adolescence extending into the early twenties, which are increasingly relevant for research and early intervention.

R#2.4: Thank you for pointing this out. We have now removed discussion of trends from the manuscript.

We are grateful for Reviewer 2's insightful comment regarding alternative definitions of childhood and adolescence. In response to this (and related feedback from Reviewers 3 and 4 on our age categorization), we have conducted additional analyses exploring alternative definitions of childhood and adolescence. Specifically, we repeated the sensitivity analyses using age cut-offs at 21 and 24 years. These analyses produced a very similar pattern of results for both the moderation analyses and the interaction models between age and follow-up interval. Notably, when applying these broader definitions of adolescence (and incorporating the additional studies identified through our updated search), the sensitivity analyses excluding the two studies that might have reflected new experiences in childhood between the two assessments remained significant for the interaction models focusing on baseline age and the interaction between baseline age and follow-up interval (page 8). This strengthens the original findings based on the age 18 cut-off and suggests that the previous non-significant result may have been due to limited statistical power

(e.g., limited number of studies undertaken in sample <18 years). We have added descriptions of these additional analyses to the Methods (page 6), Results (page 8), Supplement (pages 10–13 and 22–24), and Discussion (page 10).

Q#2.5: Conclusions
The conclusions are generally supported by the data, but some interpretations risk overstating clinical implications. While the stability of maltreatment measures is confirmed, presenting the results as directly informing clinical practice may oversimplify the issue. There is an overreliance on reappraisal as a mechanistic explanation for stability, which is then used to justify therapeutic approaches (e.g., trauma-focused CBT). Other evidence-based interventions, such as EMDR or imagery rescripting, are also relevant, and the study does not distinguish between mechanisms, so it is premature to make specific intervention recommendations. In fact, more research is needed to clarify the cognitive mechanisms underlying the stability of self-reported maltreatment – this isn't highlighted; suggestions for future avenues would be useful. There are also potential risks of overfocusing on maltreatment experiences, particularly in youth, where recall and appraisals are highly malleable. It is unclear whether the authors are implying that all young people with a maltreatment history should receive reappraisal-focused interventions, even when the primary clinical presentation is unrelated, such as panic disorder or OCD.

R#2.5: We thank Reviewer 2 for these thoughtful comments regarding the interpretation of our findings. We have now expanded the Discussion section to provide a clearer rational for the potential clinical relevance of our findings, outlining the neurobiological and cognitive processes that may render early traumatic memories comparatively unstable and modifiable. This addition further explains why therapeutic interventions may influence how such memories are retrieved, elaborated, and reconsolidated. We expanded the list of trauma-focused interventions briefly mentioned, and hope that the above discussion of mechanisms also clarifies the rationale for suggesting that trauma-focused interventions delivered early in life may have greater potential to shape how traumatic memories are incorporated in identity development, possibly mitigating risk for later psychopathology.

We also agree that more research is needed to clarify the cognitive mechanisms underlying stability and have added text on page 14 noting that "more research is needed to better understand the developmental mechanisms underlying age-related differences in the stability of childhood maltreatment reports".

In response to Reviewer 2's comment regarding whether trauma-focused interventions should be offered to those with maltreatment histories even when the primary presentation is unrelated, we note that reports of childhood maltreatment are associated with a broad spectrum of psychopathology beyond PTSD (e.g., Danese & Widom, 2020, Nat Hum Behav). Evidence indicates that trauma-focused interventions can reduce other types of psychopathology (e.g., anxiety and depression) in trauma-exposed youth even in the absence of PTSD (e.g., De Haan et al., 2024, Lancet Child Adolesc Health), suggesting that trauma-related mechanisms may contribute to diverse clinical presentations. Trauma-related memories and appraisals may therefore represent a meaningful transdiagnostic treatment target, particularly because maltreated youth consistently show poorer treatment response, more severe clinical presentations, and greater relapse risks across disorders compared to non-maltreated peers (Nanni et al., 2012 Am J Psych; Agnew Blais & Danese, 2016, Lancet).

Q#2.6: Suggested improvements
Future revisions could clarify the choice of quality assessment tools and provide justification for time intervals used in analysis. The literature search should be updated as it is almost 2 years since the search. Considering alternative definitions of adolescence and reflecting on the implications of malleability for both research and practice beyond the clinic (eg social care, legal contexts) would strengthen the manuscript, as the issue of 'stability' is perhaps more relevant there. A more balanced and open perspective on underlying mechanisms (and therefore the treatment implications) would be recommended.

R#2.6: We thank Reviewer 2 for highlighting the importance of updating our search. We have now re-run the search from 1 January 2024 to the present and identified three additional studies, which have been incorporated into the meta-analysis. All results reported now reflect the inclusion of these new studies. The overall meta-analytic estimate remained unchanged, and moderation results were largely consistent, although follow-up age reached statistical significance. Where relevant, we have updated the manuscript to reflect and discuss these revised findings.

Q#2.7: Clarity and context
The manuscript is clearly written, with an informative abstract and introduction. The rationale for the study is well justified, and the methodology is presented in detail. However, the discussion could better contextualise findings within a wider range of applied contexts where the measurement stability issue is perhaps more salient.

R#2.7: We thank Reviewer 2 for this positive evaluation. As noted in our response to Q#2.2, we have now expanded our discussion on page 14 to discuss the implications of our findings within legal and social care settings, where questions about the stability of maltreatment reports are particularly salient.

Reviewer #3

Q#3.1: This manuscript presents a comprehensive meta-analysis on the temporal stability of retrospective self-reports of childhood maltreatment across 46 studies. The topic is relevant, the data source appropriate and the manuscript is well-written and provides valuable insights. While the topic is not entirely novel, the scope and synthesis are original and significant.
While I overall commend the authors to their approach, I think the manuscript needs revision in a number of aspects to improve precision, transparency, and appropriateness of interpretation. Please note that I am not an expert in the methodology of meta-analysis, such that I advise the editor to additionally invite a reviewer with explicit expertise in this field.

R#3.1: We thank Reviewer 3 for her positive evaluation of the relevance of the topic and for her thoughtful and detailed review of the manuscript.

Q#3.2: Abstract
Line 42: The claim that "these findings challenge the prevailing view that retrospective self-reports are inherently unstable" should be substantiated. It is unclear whether this view is truly prevailing.

R#3.2: We thank Reviewer 3 for highlighting this point. As we have limited space in the abstract to substantiate this claim, we have removed the word 'prevailing' and instead focus on the assumption of their instability in the introduction.

Line 44: The statement that "reshaping trauma-related appraisals may require deliberate intervention and might be most effective during childhood" appears like a wide stretch. Please clarify how this conclusion is derived from the presented results.

We thank Reviewer 3 for the opportunity to clarify this point. In our study, we observed that trauma-related recall was highly stable over a 2.5-year period in observational cohorts, the vast majority of which did not involve trauma-focused interventions. This stability suggests that trauma-related appraisals and memories are unlikely to change spontaneously in the absence of targeted support.

We also found that stability increases with age (is significantly lower in childhood), suggesting that spontaneous change is more likely in childhood, which may identify a particularly malleable window for modifying trauma-related appraisals.

The need to reshape trauma-related appraisals emerges from our previous work showing that retrospective recall is robustly associated with psychopathology both cross-sectionally (Baldwin et al., 2025, JAMA Psych) and prospectively (Danese & Widom, 2023, JAMA Psych).

We have expanded the Discussion section in the main text of the revised manuscript draft with further focus on mechanisms, which we hope provides the readers with further context to understand the conclusions.

Q#3.3: Introduction
Line 71: Please clarify what is meant by maltreatment being a "modifiable" risk factor.

R#3.3: We thank Reviewer 3 for the opportunity to expand on this point. The distinction between modifiable and non-modifiable is often made in epidemiology and prevention science to discriminate between factors whose risk can be reduced through targeted interventions. In this case, childhood maltreatment is a 'modifiable' risk factor for poor mental health because interventions targeting maltreated children have the potential to reduce the risk of poor mental health, as independently highlighted in the highly-cited review by Arango et al. This is different, for example, from the risk linked to DNA sequence.

Line 104: The mechanisms proposed to explain higher stability in clinical samples (e.g., mood congruency, rumination, causal search bias) are mentioned but not sufficiently explained. Consider integrating the more detailed rationale from the discussion section here.

To avoid repetition between Introduction and Discussion sections, we have listed key potential mechanisms in the Introduction section and then provided details in the Discussion section, where mechanisms can be better integrated and interpreted in light of our findings.

Q#3.4: Methods
Please explain why interrater agreement was measured using percentage overlap instead of using common metrics for interrater agreement.

R#3.4: We thank Reviewer 3 for highlighting this point. We have replaced percentages with kappa values to describe interrater agreement for title/abstract and full text screening.

There is no need to mention author status or prior experience (e.g., “doctoral student”).

We have now deleted ‘doctoral student’ from the description.

From line 168 onwards, variables are listed as “e.g.”. Please provide a complete list of all extracted variables and all analyses conducted.

Thank you for highlighting this. We have removed “e.g.” as all lists are complete.

The analysis of psychopathology type as a moderator appears in the discussion but is not described in the methods. Please include it and clarify whether it was preregistered.

We are grateful to Reviewer 3 for pointing out this omission. These analyses were pre-registered, and a description of them has been added to the Methods on page 5.

The exclusion of two studies due to potential new experiences in under-18 participants should be considered for all analyses (instead of just the moderator analyses). Alternatively, you could test whether the analyses yield different results without these two studies and report as robustness checks in the supplement.

We are grateful for the opportunity to clarify our analytical strategy. We have included in the analyses a broad array of relevant studies to ensure generalizability of the findings. We have then undertaken sensitivity analyses to ensure that the findings are not biased by study selection. In particular, we have identified two (out of 49) studies where there was some ambiguity over whether the assessment of childhood maltreatment focused on the same experiences at baseline and follow-up (the studies did not explicitly specify the same reporting period across time points, and the follow-up assessment might have included maltreatment experiences that occurred after the baseline assessment). We have rerun the main analyses as well as the meta-regression analyses testing the effects of baseline age and follow-up interval duration. Such sensitivity analyses found similar results to the analyses including all studies (page 8).

The dichotomization of age at 18 years seems problematic to me. This cutoff lacks theoretical justification (e.g., regarding cognitive processes) and reduces information. If retained, mark it clearly as exploratory and not preregistered. Also, please clarify the age distribution within the 0–18 group (e.g., proportion of children vs. adolescents).

We thank Reviewer 3 for this thoughtful comment. We acknowledge that the dichotomization of age at 18 years was post-hoc and have now explicitly stated this in the Methods (page 6). We also provide a stronger theoretical rationale for the choice of this cut-off, noting on page 6 that autobiographical memory undergoes major developmental changes from adolescence to adulthood, which could plausibly affect the stability of recall.

In addition, in response to comments from Reviewer 3 (and other reviewers) regarding the classification of age groups, we conducted additional sensitivity analyses with different age cut-offs to further assess the robustness of our findings. Specifically, we explored alternative definitions of adolescence using age cut-offs at 21 and 24 years and repeated the moderation analyses. These additional analyses produced consistent results. Details of these analyses are provided in the Methods (page 6), Results (page 8), Supplement (pages 13–16 and 27–29), and Discussion (page 10).

Q#3.5: Results
Descriptive statistics for key variables (e.g., maltreatment types, study quality) should be included in the main text, not only in the supplement.

R#3.5: All key variables in each study and across studies are described in the tables and the initial paragraph of the Results section (page 7). The derivation of the study quality variable is detailed in the Supplementary Materials to comply with the word count limit.

Age should be described more thoroughly: include range, median, and proportion of participants under 18 and/or show the distribution visually.

We thank Reviewer 3 for this suggestion. We have added range and median for baseline age in the description of key variables in the initial paragraph of the Results section (page 7). The proportion of different age groups is reported in the moderation table (Table 2). A display of the age distribution is in Supplementary Figures 6 and 7.

Line 242: Please explain the large number of effect sizes (224) relative to the number of studies (46). Clarify what these effect sizes represent.

We thank Reviewer 3 for the opportunity to clarify this point. Several studies contributed multiple effect sizes, for example, reporting separate estimates for different maltreatment types or for subgroups within the sample (e.g., males

versus females, patients versus controls). This resulted in a larger number of effect sizes than studies. To account for the non-independence of these estimates within studies, we used a multilevel meta-analytic model. We have added clarification of this point to the Methods section on page 6.

Justify the use of correlation coefficients (r) instead of standard test-retest reliability measures. It would be helpful if you also report the effect size metric used in the original studies.

The decision to harmonize effect sizes onto the correlation scale was guided by the data, which were overwhelmingly reported on the correlation scale. 63% of all effect sizes reported from studies were either Pearson's, Spearman's, or Intraclass correlation coefficients. A further 23% reported 2x2 tables where tetrachoric correlations could be easily calculated, leaving only 14% of studies where tetrachoric correlations had to be estimated from kappa values using the calibration model. A description of this has been added to the data harmonization section of the Supplementary Materials on page 8.

Please additionally include a more interpretable measure such as percentage overlap (between the two time points), especially since percentage overlap is already partially used in figures and the discussion (e.g., line 318), but lacking in the results section. Also report omission vs. commission rates in the results section to better understand the non-overlapping part of the reports.

We are thankful to Reviewer 3 for this suggestion. A discussion of the Sankey plot results and how the percentage overlap was derived has been added to the results on page 7.

Ensure consistent reporting of statistical parameters across moderator analyses (e.g., neglect vs. abuse, representative vs. non-representative samples).

We are grateful for the opportunity to clarify this point. We reported details of all pre-registered moderation analyses in Table 2 and provided key statistics (*Q* statistic and corresponding *p*-values) in the main text. For post-hoc moderation analyses, we reported detailed statistics in the main text.

Avoid reporting "trends for significance." Please use a predefined significance threshold and stick to it.

We have now removed discussion of trends from the manuscript.

Clarify which analyses were preregistered and which were added post hoc (e.g., logarithmic vs. linear age terms).

Thank you for highlighting this. We have now clarified in the Methods section (page 6) and the Results section (pages 7-9) which analyses were post-hoc.

Line 283: Please provide inferential statistics for the comparison between younger and older participants.

The key test statistics for the group comparisons are the *Q* statistic and corresponding *p*-values, which are reported throughout the text.

Line 291: Report the effect size, not just the p-value, for the interaction term.

We thank Reviewer 3 for pointing this out. We have now added the effect size for the interaction term.

Table 2: Clarify the meaning of the correlation column for continuous moderators (e.g., age, sex, attrition).

A note has been added to clarify the meaning of the correlation column for continuous moderators on page 20.

Table 3 is not referenced in the text. Please integrate and explain.

Thank you for the opportunity to clarify this. Table 3 is referenced on the third paragraph of page 9.

Q#3.6: Discussion

Line 318: The “20%” divergence in reporting comes out of the blue and should be introduced earlier in the results.

R#3.6: We thank Reviewer 3 for pointing this out. We have now added a discussion of the Sankey plot and percentage overlap to the results on page 7.

Line 334: Consider whether lower certainty about experiences may contribute to lower stability in population-based samples.

We are grateful to Reviewer 3 for highlighting this possible explanation, which we have added to the discussion on page 9.

Line 350: you mention that „Causal search bias, where individuals reinterpret past experiences to explain their distress or difficulties, could also increase consistency by reinforcing negative interpretations of memories over time". Could causal search bias also lead to inconsistency of reports if interpretations change over time?

The association between causal search bias – typically observed in individuals experiencing mental health difficulties – and report inconsistency is not supported by our data, which showed higher stability in clinical samples. However, Reviewer 3 raises an intriguing hypothesis--that changes in mental health could, in theory, also lead to inconsistency in reports over time. Unfortunately, there were insufficient studies reporting data on changes in mental health status to test this possibility directly. We note that this is highlighted as a limitation on page 12, where we write that we were unable to examine the effects of changes in psychopathology on report stability.

Line 355: The phrase "Of note" is ambiguous. Clarify whether these findings were preregistered.

Thanks for the opportunity to clarify this. We have now clarified in the methods that analyses related to psychopathology were pre-registered.

Line 360: The sentence "Because low stability adds to measurement error…" is unclear in its continuation. Please revise.

Thank you for this feedback. We have edited this sentence on page 10 to better explain the link.

Line 362: Clarify what neglect is being compared to in "lower agreement with prospective measures."

Thank you for highlighting this. The comparison of neglect with abuse has been clarified on page 10.

Line 363: Avoid using "on one hand… on the other hand…" when both arguments point in the same direction.

We would prefer to keep the text as we aim to further the contrast between abuse and neglect in the relevant sentences.

Line 363: The claim that abuse is a stronger emotional experience than neglect should be supported by evidence.

Thank you for highlighting this. We have edited the text to ‘threatening’ on page 10 of the revised manuscript draft – the references listed provide evidence for the statement

Line 373: Specify that “first recalled maltreatment in childhood” refers to the first measurement point of this study, not the first recall in general.

We are grateful that this has been pointed out and have now clarified that this refers to the first measurement point of the study on page 10.

Line 376: The analysis that excluded the two ambivalent studies likely provides more reliable results. Please reframe the interpretation accordingly.

As noted above, we have included in the analyses a broad array of relevant studies to ensure generalizability of the findings. We have then undertaken sensitivity analyses to ensure that the findings are not biased by study selection. Such sensitivity analyses found similar results to the analyses including all studies. The interpretation of the findings in the revised manuscript draft reflects this approach.

Line 385: „Children’s and adolescents’ memories may be more malleable and susceptible to reinterpretation ... In contrast, adults’ memories may be more stable over time because of repeated rehearsal and integration into a coherent life narrative“ - Consider discussing that on the other hand, maltreatment events are temporally more distant for adults, which may affect memory accuracy negatively.

We thank Reviewer 3 for this suggestion. To examine this hypothesis, we have tested the moderation of maltreatment recall stability by the baseline age of the cohort (a proxy for the time elapsed between exposure in childhood and assessment). The findings are that stability is higher (not lower) in cohorts of adults vs children – however defined –, and, as such, the hypothesis is not supported by the data.

Line 390: The comparison to personality traits and cognitive abilities does not seem very informative or appropriate to me. These constructs (abilities and traits) differ fundamentally from event memory. (also see line 411). CSA experiences should not be treated as a personality trait.

We appreciate the opportunity to clarify this point. In the text, we do not treat child sexual abuse as a personality trait. Instead, we highlight that the memory system – and here particularly maltreatment memories – show striking similarities to other key psychological functions (personality and cognition) in that all systems appear less stable, or more malleable, in childhood and adolescence vs adult life.

Line 398: "„The findings should also be interpreted within the context of research studies similar to those included in the meta-analyses and may not generalise to other contexts (e.g., to disclosures that reach the attention of authorities)." Please expand on generalizability. Specify which contexts the findings apply to and how these differ from cases reported to authorities.

We appreciate this feedback and have added a further description on page 11.

„While no significant effect was detected in our moderation analysis, attrition could still introduce bias" – Please mention the potential direction of bias here.

We thank Reviewer 3 for this suggestion. We have added explanatory text on page 11 of the revised manuscript draft.

Line 451: The therapeutic implications are not clearly supported by the data. Please clarify the connection and avoid overinterpretation. Also: Why should interventions be more usefull in childhood, if reports have shown to be more inconsistent in childhood?

In response to this comment by Reviewer 3, we have clarified the therapeutic implications in the concluding section (page 13) by expanding the text to outline the neurobiological and cognitive processes that may render early traumatic memories comparatively less stable and more modifiable. These developmental characteristics provide a theoretical rationale for why therapeutic interventions may more readily influence how early memories are retrieved, elaborated, and consolidated, as the greater malleability of memory representations in childhood and adolescence may offer an important window for shaping how traumatic experiences are processed and integrated in identity development and affect risk for later psychopathology.

Q#3.7: General Comments
Clarify how original studies handled ambivalent or uncertain reports, e.g., participants reporting being unsure about having experienced maltreatment. How were they forced into a dichothomeous variable? Was certainty about responses measured?

R#3.7: Reviewer 3 raises an interesting conceptual point about levels of certainty; however, this was unfortunately not something that the original studies measured or reported on. As such, we are unable to address this hypothesis in our meta-analysis.

Did you examine potential changes of maltreatment type between the two timepoints? (i.e., stable overall report but changing subtype)?

We thank Reviewer 3 for the opportunity to clarify this point. Where possible, we extracted stability estimates by subtype as well as overall measures (e.g., stability of reporting one specific type of abuse over time). Most studies reported individual subtype stability estimates, as listed in the study characteristics table. However, studies have not examined changes in maltreatment types over time, and this could, therefore, not be examined in our meta-analysis.

The interpretation of r = .79 as "very stable" should be contextualized. (even a correlation of r = .62 is considered „high stability", line 397). I would appreciate a more differeniated interpretation. What level of stability is expected by yourself, by lay people or by experts? How does this compare to stability of other memory constructs?

We thank Reviewer 3 for this suggestion. We based our qualitative description of the correlational effect sizes on conventions suggested by Cohen, which identifies r>.5 as "large" (Cohen, 1988). We have now clarified this in the supplementary Methods section. There is no further conventional distinction for r>.5. As such, we cannot provide a clear differentiation. Inspired by the suggestion from Reviewer 3, we have added some text to the Discussion section to contextualise the observed effect size in light of results on the stability of memories for broader measures of childhood experiences and other key psychological constructs on page 9 of the revised manuscript draft.

Discuss limitations of dichotomous classification (maltreatment vs. no maltreatment) in terms of memory and psychometrically / statistically.

We thank Reviewer 3 for this helpful comment. While some studies used dichotomous classifications of maltreatment (e.g., maltreatment vs no maltreatment), not all measures were binary. Our meta-analysis combined effect sizes on the correlational scale, including tetrachoric, intraclass, Pearson, and Spearman correlations, which together captured both continuous and binary measures of maltreatment. In response to this comment, we conducted additional analyses testing whether the type of measurement scale (binary vs continuous) moderated stability estimates. We found no significant differences, suggesting that the overall findings are not driven by the use of dichotomous versus continuous measures.

Table 3 shows no effect of interventions on memory stability. Could this challenge assumptions about repression and memory recovery in therapy? This would be worth mentioning.

Reviewer 3 raises an interesting point about the potential implications of our findings in challenging assumptions about repression and memory recovery in therapy. In the last of the limitations listed in the Discussion section, we note that only 6 of the included studies examined changes potentially linked to an intervention, and only one study where the intervention was trauma-focused, i.e., specifically targeting maladaptive cognitive appraisals or memory processes (Hoeber et al., 2025). Because of the very limited information available, we opted to avoid directly commenting on issues related to repression and memory recovery in therapy.

Reported maltreatment experiences are treated as „memories“ in the manuscript. Consider referring to “reports” rather than “memories,” and discuss the distinction between self-report and factual accuracy.

We thank Reviewer 3 for this thoughtful suggestion. Based on this comment, we have now changed “memories” to “self-reports” in the manuscript where relevant.

Line 434: I disagree with your claim that retrospective measures of maltreatment can be treated as trait-like features. We still don’t know if the reports are based on actual experiences, just because their are reported consistently (also considering former studies by your research team on inconsistent reporting).

As explained above, we do not treat retrospective measures of maltreatment as a trait-like feature. Instead, we highlight that the memory system – and here

particularly maltreatment memories – show striking similarities to other key psychological functions (personality and cognition), in that all systems appear less stable, or more malleable, in childhood and adolescence vs adult life. Based on this feedback by Reviewer 3, in the relevant text of the revised manuscript draft, we have edited "trait-like" to "stable" for clarity

I find this conclusion hard to follow: "It also suggests that the lack of agreement between prospective and retrospective measures is unlikely to be driven by shifting retrospective reports over time, and instead may be driven by stable individual differences between those identified as maltreated by each measure". I dont see how this conclusion can be drawn from your data. Reports could be consistent, but false. Please clarify.

We are grateful for the opportunity to clarify this point by Reviewer 3. First, we want to highlight an important methodological distinction. The current paper focuses on the stability of retrospective reports of childhood memory. In contrast, previous work by our team (e.g., Baldwin et al, 2019, JAMA Psychiatry) focused on the disagreement between prospective and retrospective measures of maltreatment. To clarify this distinction, we have added a sentence in the first paragraph of the Introduction section on page 3. As previously discussed (e.g., Coleman et al, 2024, JCPP), one of the reasons why disagreement between prospective and retrospective measures of maltreatment might emerge is the instability of retrospective measures – instability (different reports over time) adds to measurement error, and measurement error inevitably reduces the possible agreement between two different (i.e., prospective vs retrospective) measures. We can find some discussion of these standard psychometric notions in the Introduction section on page 3 "Low stability of retrospective maltreatment measures contributes to measurement error and misclassification" and we have now added a reference to a psychometric textbook for further discussion of this topic on pages 3 and 12 of the revised manuscript draft. In the excerpt from our manuscript that Reviewer 3 has pasted in her comment above, we highlight that this hypothesis is not supported by data from the meta-analysis: "lack of agreement between prospective and retrospective measures is unlikely to be driven by shifting retrospective reports over time". This is because of the observed stability of maltreatment reports. Instead, we suggest that disagreement between prospective and retrospective measures of childhood maltreatment "may be driven by stable individual differences" in the way people recall maltreatment experiences. To further clarify this concept, we have edited the text on page 12.

Also, how does the interpretation of „stable individual differences“, relates to the findings of your former studies on inconsistent reporting of maltreatment between childhood and adulthood? As an alternative explanation, there may be inconsistent reporting during childhood until people stick to their report, irrespective of truth-status.

Please see above

Your work would overall benefit if you discuss your findings more in light of other work by Danese and colleagues on inconsistencies between prospective and retrospective reports, and between childhood and adolescent reporting.

We thank the Reviewer for her interest in our previous work. We believe we have adequately discussed our previous work on the disagreement between prospective and retrospective measures of maltreatment – both in the Introduction and the Discussion sections. Please see above. We prefer not to extend the discussion on the related but separate issues of disagreement to maintain conceptual focus within the paper.

I also think you could somewhat enrich the introduction section. Maybe my former comments provide inspiration for further arguments to include. But this is probably more a matter of taste.

We thank Reviewer 3 for the extremely thoughtful, helpful, and details suggestions, which we believe have significantly improved the revised manuscript draft. We made an addition to the text in the Discussion section and opted not to add further text in the Introduction section to avoid repetition.

Thank you for the opportunity to review this manuscript.
Elsa Gewehr

<u>Reviewer #4:</u>

Q#4.1: In the manuscript the authors report a systematic review on a highly relevant and interesting area. The meta-analysis includes 46 studies (37,880 participants) investigating the temporal stability of retrospective self-reports of childhood maltreatment. The authors report an overall high stability over an average 2.5-year interval, while noting considerable heterogeneity across studies. Stability varied by maltreatment type (neglect < abuse), sample type (population-based < clinical), and age (lower in younger participants, particularly under 18 years). In my opinion, this study addresses an important gap in the literature and provides knowledge with clear implications for both research methodology and clinical practice. The study comes with several strengths, which I will list below. However, I also have some concerns and suggestions which I believe, if taken into account, will improve the manuscript.

Strengths:

-Novelty and significance: To my knowledge, this is the first quantitative synthesis of retrospective
-The study comes with rigorous methodology.
-Comprehensive analyses: The use of multilevel models, sensitivity test, and moderation analyses is impressive and allows nuanced interpretation.
-Clarity of reporting: The manuscript is well structured, with transparent methods and clear figures.

R#4.1: We appreciate Reviewer 4's positive evaluation of the manuscript, including its methodology, analytical approach, and implications.

Major concerns/suggestions:

Q#4.2: Conceptual framing: The introduction could and should more explicitly express how findings inform the validity of retrospective self-reports as measures of exposure vs. memory accuracy. With this I particularly mean: Are we interpreting stability as evidence for reliability of reports, or as insights into memory processes? Clarifying this conceptual distinction would strengthen the discussion of the results.

R#4.2: We thank Reviewer 4 for this suggestion. To clarify the conceptual framing, we have added a sentence in the Introduction section (page 3) to highlight our previous work on disagreement between prospective and retrospective measures and separate it from the key research question of the current paper. Instability (low test-retest reliability) might have contributed to disagreement between prospective and retrospective measures (by increasing

measurement error), but the findings of high stability in recall are against this hypothesis. However, instability should not be taken as an indicator of validity, as the prospective measures are also biased in several important ways and do not provide a 'true' measure of maltreatment. We discuss the relationship between reliability and disagreement in the Discussion section on page 10, but we opted not to add more details here as this is not the key focus of the paper, and more details early in the paper would likely be confusing for readers.

Q#4.3: Interpretation of heterogeneity: The I^2 value is extremely high (>98%). While moderation analyses identify some meaningful moderators, residual heterogeneity remains substantial. It would be useful for the authors to discuss whether this reflects measurement variability, true differences in recall processes, or both.

R#4.3: We thank Reviewer 4 for highlighting the issue of heterogeneity. Inspired by this comment, we have expanded the first paragraph in the Discussion section (page 9) to better highlight [1] the implication on study heterogeneity (a cautious interpretation of the overall meta-analytical effect size) and [2] the rationale of moderation analyses to identify factors explaining study heterogeneity. The moderation analyses focus on separate hypotheses (akin to bivariate analyses), and it is unclear how large the multivariate residual (unexplained) heterogeneity might be. What we do know is that several of the identified moderators individually explain a sizeable proportion of the variance.

Q#4.4: Age/developmental issues:
1)The age x interval interaction is one of the most compelling findings, yet the main text presents it rather briefly. The introduction should address developmental theories on autobiographical memory (e.g., narrative identity, recall of traumatic periods/events, consolidation) to a greater extent. (See e.g., Bauer et al., Hjuler et al., Fivush et al.,). This is essential to ground the study results more strongly in cognitive developmental science.
2) When referring to age >18, the authors write 'children', however, the WHO definition of children over 10 years of age is 'adolescents'. I recommend that you consider which term to use, and at least to explain what is meant by 'children' in your study.
3) A major concern is also that the authors cut-off when referring to children is 18 years of age. Considering the massive development on autobiographical development during childhood (with regards to encoding, consolidation and recall), it would be valuable to consider a lower-age cut-off. For instance 12-15-years of age; 15-18-years of age and above 18 years of age.

4) In line with the above; the authors discuss ‘re-shaping trauma related memories in childhood’. However, with a cut-off of 18-years of age, such a discussion should be considered carefully.

R#4.4: We thank Reviewer 4 for their insightful comments on age and memory stability. We have added further detail on developmental theories of autobiographical memory – including cognitive and neurobiological processes – to the Introduction (page 3) and expanded on these theoretical perspectives in the Discussion section focusing on clinical implications on page 13. In response to these points (and related feedback from other reviewers) on age categorization, we have also conducted additional sensitivity analyses and expanded the Discussion section to reflect the updated results. Details of these analyses are provided in the Methods (page 6), Results (page 8), Supplement (pages 13-16 and 27-29), and Discussion (page 10). We also agree that finer-grained age distinctions (e.g., 12–15 years) could provide valuable insights; however, this was not possible in the present analyses due to the small number of studies in this range (k=3), which precluded meaningful moderation testing.

Q#4.5: Time interval and measure types:
1) The study includes studies with a time interval of two months or longer. What is the rationale for this time interval? This must be explained in the manuscript.

R#4.5: We thank Reviewer 4 for the opportunity to clarify this. On page 5, we highlight the decision to include only studies with a time interval of two months or longer, which was a deviation from the study’s original pre-registration plan:

“This decision was guided by our interest in examining the stability of maltreatment memories over time rather than the short-term test-retest reliability of maltreatment measures; this resulted in the exclusion of k=54 studies assessing the psychometric properties of maltreatment measures being developed.”

Q#4.6: 2) In addition, the authors write that ‘for studies that measured stability at multiple time points, the longest interval was selected by default’, Please explain the rational behind this choice of selection as well.

R#4.6: We are grateful to Reviewer 4 for drawing our attention to this. The rationale for selecting longer intervals, where possible, was guided by our interest in examining memory-related processes, which are more likely to be

detected over longer intervals. We have added a description of this rationale on page 5.

Q#4.7: 3) While interviews vs questionnaires did not moderate stability, could measure length/detail (e.g., single-item vs. multi-item scales) be a relevant factor? If such data are unavailable, this limitation should be acknowledged.

R#4.7: Reviewer 4 raises an important point regarding measure length and detail as a potential moderating factor. Inspired by this comment, we conducted a post-hoc moderation analysis within the subset of studies using questionnaires to examine whether single- versus multi-item measures differed in stability. We observed a trend towards higher stability for multi-item questionnaires, whereby greater measurement detail might contribute to more consistent reporting. A description of this analysis has been added to the results (page 8) and discussion (page 9)

Q#4.8: Neglect vs. abuse: The finding of lower stability for neglect deserves further unpacking. The authors suggest emotional salience as an explanation, but neglect also differs conceptually (absence of care rather than presence of harmful acts). This distinction could be elaborated with reference to how neglects is operationalized and experienced. Also, when explaining the different types of child maltreatment, examples could be helpful. Please make sure to provide sufficient references.

R#4.8: We thank Reviewer 4 for the opportunity to clarify this point. In the manuscript, we highlight on page 10 the different conceptualizations of neglect versus abuse that may contribute to differences in stability:

“On the other hand, neglect involves the absence of action or resources, rather than the occurrence of more tangible events that typically serve to anchor memories, and its definition might vary over time as individuals and societies change their interpretations of what constitutes a child’s ‘basic needs’ and adequate caregiving practices.”

Q#4.9: Clinical implications: The discussion highlights risks of misclassification, but clinical applications (e.g., use in assessment, legal testimony, trauma-informed care) could be more directly addressed. How should practitioners interpret a high overall stability yet 20% change in reports?

R#4.9: We thank Reviewer 4 for this important point. In response, we have added further discussion on the relevance of maltreatment report stability and accuracy

within legal and social care contexts (page 12-13), and we have also provided additional contextualization of the clinical implications of our findings (page 13). However, given the differing views raised by reviewers regarding the extent to which these implications should be emphasized, we have not expanded this section further in order to balance these perspectives.

Minor Comments

Q#4.10: 1) Some references are duplicated in the text (e.g., Coleman et al., 2024 cited twice in the same line). A careful proofread is needed.

R#4.10: We thank Reviewer 4 for pointing this out and have removed this duplicated reference and carefully proofread throughout.

Q#4.11: 2) Figures and supplementary tables are well designed, but figure legends could briefly restate the key finding for accessibility.

R#4.11: Figure legends in the main text now all include further text restating the key findings.

Q4#.12: 3) In the methods, clarify whether quality ratings were incorporated into sensitivity analyses or only descriptively reported.

R#4.12: In the methods on page 6, we state that we used meta-regression to test whether stability was moderated by a set of a-priori defined factors, including study quality.

Q4#.13: 4) Reporting of participant demographics: if available, indicate diversity (ethnicity, socioeconomic background) across studies, as these may shape recall stability.

R4#.13: We thank Reviewer 4 for raising this important point. Unfortunately, participant demographics such as ethnicity and socioeconomic background were not consistently reported across studies, and, therefore, we were unable to conduct moderation analyses testing their effect on report stability. We have now added a discussion of this in the limitation section on page 12.

Q#4.14: 5) Wording: the phrase “non-negligible proportion (20%)” could be rephrased for clarity (“approximately one in five participants changed their report”).

R4#.14: We have now rephrased this sentence as suggested for clarity.

Q#4.15: 6) Please provide kappa values when reporting inter-rater agreement results.

R#4.15: We have replaced percentages with kappa values to describe interrater agreement for title/abstract and full text screening.

Q#4.16: 7) Line 175: Please explain the rationale. ("For studies that measured stability at multiple time points, the longest time interval was selected by default")

R#4.16: We have added a sentence explaining that the longest time interval was selected based on our interest in examining memory-related processes, which are more likely to emerge and become more pronounced over longer periods.

Q#4.17: 8) Line 395: "The fining should be interpreted….." I believe a 's' is missing in 'finding'?

R#4.17: Thank you for highlighting this. We have now corrected this error.

Q#4.18: 9. Line 395: "provides reassurance" – I would recommend to write this more cautiously.

R#4.18: Thank you for this suggestion. We have revised the phrasing to "provides some reassurance" on page 12 to convey a more cautious interpretation.

Q#4.19: 10. Line 442: When addressing that interventions might be more beneficial if delivered early in life, please keep in mind that psychiatric diagnosis because of maltreatment are typically diagnosed later in life.

R#4.19: We agree with Reviewer 4 that psychiatric diagnoses linked to maltreatment are sadly often diagnosed only later in life. Our suggestion would be to consider interventions to modify maltreatment-related appraisals early in life in order to potentially prevent or mitigate the risk of later psychiatric diagnoses; we also implicitly suggest that more trauma-focused interventions should be considered and evaluated, even for psychiatric disorders beyond PTSD (e.g., depression, anxiety).
